# The OxyR and SoxR transcriptional regulators are involved in a broad oxidative stress response in *Paraburkholderia xenovorans* LB400

**DOI:** 10.1186/s40659-022-00373-7

**Published:** 2022-02-20

**Authors:** Valentina Méndez, Laura Rodríguez-Castro, Roberto E. Durán, Gabriel Padrón, Michael Seeger

**Affiliations:** 1 Laboratorio de Microbiología Molecular y Biotecnología Ambiental, Departamento de Química and Centro de Biotecnología Dr. Daniel Alkalay Lowitt, Universidad Técnica Federico Santa María, Avenida España, 1680 Valparaíso, Chile; 2 Centro de Ingeniería Genética y Biotecnología, Ave. 31 No. 15802, Playa Havana, Cuba

**Keywords:** Oxidative stress, *Paraburkholderia xenovorans*, Paraquat, Hydrogen peroxide, Superoxide, OxyR, SoxR

## Abstract

**Background:**

Aerobic metabolism generates reactive oxygen species that may cause critical harm to the cell. The aim of this study is the characterization of the stress responses in the model aromatic-degrading bacterium *Paraburkholderia xenovorans* LB400 to the oxidizing agents paraquat and H_*2*_O_2_.

**Methods:**

Antioxidant genes were identified by bioinformatic methods in the genome of *P. xenovorans* LB400, and the phylogeny of its OxyR and SoxR transcriptional regulators were studied. Functionality of the transcriptional regulators from strain LB400 was assessed by complementation with LB400 SoxR of null mutant *P. aeruginosa* ΔsoxR, and the construction of *P. xenovorans* pIZ*oxyR* that overexpresses OxyR. The effects of oxidizing agents on *P. xenovorans* were studied measuring bacterial susceptibility, survival and ROS formation after exposure to paraquat and H_*2*_O_2_. The effects of these oxidants on gene expression (qRT-PCR) and the proteome (LC–MS/MS) were quantified.

**Results:**

*P. xenovorans* LB400 possesses a wide repertoire of genes for the antioxidant defense including the *oxyR*, *ahpC*, *ahpF*, *kat*, *trxB*, *dpsA* and *gorA* genes, whose orthologous genes are regulated by the transcriptional regulator OxyR in *E. coli*. The LB400 genome also harbors the *soxR, fumC*, *acnA*, *sodB*, *fpr* and *fldX* genes, whose orthologous genes are regulated by the transcriptional regulator SoxR in *E. coli*. The functionality of the LB400 *soxR* gene was confirmed by complementation of null mutant *P. aeruginosa* Δ*soxR*. Growth, susceptibility, and ROS formation assays revealed that LB400 cells were more susceptible to paraquat than H_2_O_2_. Transcriptional analyses indicated the upregulation of the *oxyR*, *ahpC1*, *katE* and *ohrB* genes in LB400 cells after exposure to H_2_O_2_, whereas the *oxyR*, *fumC*, *ahpC1*, *sodB1* and *ohrB* genes were induced in presence of paraquat. Proteome analysis revealed that paraquat induced the oxidative stress response proteins AhpCF and DpsA, the universal stress protein UspA and the RNA chaperone CspA. Both oxidizing agents induced the Ohr protein, which is involved in organic peroxide resistance. Notably, the overexpression of the LB400 *oxyR* gene in *P. xenovorans* significantly decreased the ROS formation and the susceptibility to paraquat, suggesting a broad OxyR-regulated antioxidant response.

**Conclusions:**

This study showed that *P. xenovorans* LB400 possess a broad range oxidative stress response, which explain the high resistance of this strain to the oxidizing compounds paraquat and H_2_O_2_.

**Supplementary Information:**

The online version contains supplementary material available at 10.1186/s40659-022-00373-7.

## Introduction

Aerobic metabolism induces partial oxygen reduction, generating reactive oxygen species (ROS: ${\text{O}}_{2}^{ - }$, OH· and H_2_O_2_). Formation of ROS may cause critical harm to the cell. However, antioxidant mechanisms of the cell counteract the toxicity of ROS. The transcriptional regulators OxyR and SoxRS from *Escherichia coli* respond to oxidative stress caused by hydrogen peroxide (H_2_O_2_) and superoxide (${\text{O}}_{2}^{ - }$), respectively, activating the expression of diverse antioxidant enzymes (e.g., superoxide dismutase, alkyl hydroperoxide reductase, catalase). In *E. coli*, the OxyR transcriptional regulator is commonly activated by H_2_O_2,_ while SoxR is activated by superoxide producing redox-cycling compounds, such as paraquat (PQ; methyl viologen) and phenazine methosulfate (PMS) [29]. In non-enteric Proteobacteria and Actinobacteria such as *Pseudomonas* and *Streptomyces* strains, SoxR is activated by endogenous redox-active compounds (e.g*.*, phenazine, actinorhodin) or their precursors, and exogenous redox compounds such as paraquat [22, 30]. Therefore, SoxR mediates primarily the response to redox-cycling molecules but not to superoxide. However, the DNA-binding property of SoxR is conserved in enteric bacteria and non-enterics [80]. In non-enteric bacteria, the SoxR may regulate the antibiotic production and export, the oxidative stress response and the morphological development [22, 30, 80].

Oxidative stress and ROS accumulation in the cell may be enhanced by environmental factors, such as the presence of aromatic compounds, heavy metals and quaternary ammonium compounds [2, 3, 17, 43, 51, 61, 67]. *Paraburkholderia xenovorans* LB400^T^ (previously classified as *Burkholderia xenovorans* LB400) is a model and versatile aromatic-degrading bacterium that has been widely studied [14]. The metabolism of aromatic compounds in strain LB400 induces general and oxidative stress and may produce toxic compounds [2, 3, 9, 16, 48, 61]. Oxidative stress is detrimental for aromatic biodegradation [15, 61]. Notably, the addition of antioxidant compounds such as α-tocoferol enhances the degradation of chlorobiphenyls by *P. xenovorans* LB400 [61]. Mechanisms involved in *P. xenovorans* LB400 response to oxidative stress caused by oxidizing agents are barely known. The aim of this study is the characterization of the stress responses of the model aromatic-degrading bacterium *P. xenovorans* LB400 to the oxidizing agents paraquat and H_*2*_O_2_.

## Materials and methods

### Chemicals

Paraquat dichloride hydrate (PQ; > 99% purity) and phenazine methosulfate (PMS) were obtained from Sigma-Aldrich (Saint Louis, MO, USA) and MP Biomedicals (Irvine, CA, USA), respectively. Hydrogen peroxide (H_2_O_2_) solution (9% v/v) was obtained from Diphem Pharma S.A. (Santiago, Chile).

### Bacterial strains and culture conditions

*P. xenovorans* strains were cultivated in modified Luria–Bertani at 30 °C or in M9 mineral medium with trace solutions and glucose (5 mM) or biphenyl as the sole carbon and energy source [50]. For recombinant *P. xenovorans* strains, gentamicin (10 µg µl^−1^) was added. The effect of oxidizing agents on *P. xenovorans* growth was assessed by adding different paraquat and H_2_O_2_ concentrations to exponential-growing cells (turbidity at 600 nm of 0.5) in M9 mineral medium with glucose (5 mM). *E. coli* S17λpir was grown in LB medium at 37 °C. *Pseudomonas aeruginosa* cells were cultivated in LB or M63 minimal media at 37 °C. Gentamicin (10 µg µl^−1^) was added for recombinant *P. aeruginosa* strains. Bacterial growth in liquid media was determined by measuring turbidity at 600 nm.

### Bioinformatic analyses

To search for stress response genes in *P. xenovorans* LB400 genome, sequences of proteins from several bacteria were retrieved from protein databases (UniProt, NCBI). For the LB400 genome analyses, two databases were employed (http://genome.ornl.gov/microbial/bfun/ and http://www.burkholderia.com/). BLAST (BLASTP and TBLASTX) search tools [4] were used to compare query sequences with the LB400 genome. Swiss-Prot protein database was employed for protein searches [5]. An amino acid sequence identity ≥ 30% was used for protein identification.

### Phylogenetic analysis of OxyR and SoxR transcriptional regulators

Several OxyR and SoxR amino acid sequences with experimental evidence were retrieved from the UniProtKB Swiss-Prot/TrEMBL database [6]. All the amino acid sequences were aligned, using the M-Coffee server (using the following methods: Mpcma_msa Mmafft_msa, Mdialigntx_msa, Mpoa_msa, Mmuscle_msa, Mprobcons_msa, Mt_coffee_msa) [52]. Ambiguous positions were trimmed using the gappyout strategy of the TrimAl software [11]. The MSA were evaluated using the editor AliView version 1.24 [40]. The best partitioning scheme was identified, using the program PartitionFinder version 2.1.1 [39]. A distribution of probable trees was obtained, by Bayesian Inference as implemented in MrBayes 3.2.6 [64]. Two separate runs of 250.000 generations were executed (two chains each run,sampling every 1000 generations). Visualization and editing of phylogenetic trees were performed using the FigTree v. 1.4.2 software (http://tree.bio.ed.ac.uk/software/figtree/). The bootstrap values (percentage) > 50% were shown for each branch point.

### Molecular biology techniques

Recombinant DNA techniques were performed according to standard methods [66]. Plasmid DNA was isolated with the E.Z.N.A. Plasmid Mini Kit I (Omega Bio-tek, Norcross, USA) according to manufacturer recommendations. DNA was sequenced using an ABI Prism 377 automated DNA sequencer (Applied Biosystems Inc., Foster, CA, USA).

### Construction of *P. aeruginosa* strain ∆soxR::BxeC1217

To study the functionality of the *soxR* gene (BxeC1217) of strain LB400, a complementation assay was performed with *P. aeruginosa ∆soxR* [21] using the LB400 BxeC1217 gene. As positive control*, P. aeruginosa ∆soxR* was complemented with the PA2273 gene (*soxR*) of *P. aeruginosa* PA14. For the plasmid construction, the *P. xenovorans* LB400 BxeC1217 and *P. aeruginosa* PA14 PA2273 genes were cloned into the suicide vector pMQ30, along with a region upstream and a region downstream of the PA2273 gene of strain PA14. An identity of 28.6% between SoxR regulators of *P. xenovorans* LB400 (BxeC1217) and *P. aeruginosa* PA14 (PA2273 gene) was determined by a pairwise sequence alignment (https://www.ebi.ac.uk/Tools/psa/emboss_needle/). The recombinant plasmids were transferred to *P. aeruginosa ∆soxR* strain by conjugation using *E. coli* S17ʎpir.

### Construction of *P. xenovorans* strain pIZoxyR

A recombinant *P. xenovorans* strain overexpressing *oxyR* gene was constructed, which allowed to study the protective effect of this transcriptional regulator under oxidative stress. The *oxyR* gene (BxeB3987) was amplified by PCR from LB400 genomic DNA using the oligonucleotides oxyR-5′ (CCTCTAGAGCGCGGCCGTCAGTTGAC) and oxyR-3′ (CCAAGCTTTGCCCTAAGGAGGTAAAACATGACCCTCACCGAACTGAAATACATC). Primer oxyR-5′ contains *Xba*I restriction site, while primer oxyR-3′ contains *Hind*III restriction site. The DNA fragment was cloned in the broad-host range plasmid pIZ1016, a derivative of vector pBBR1MCS-5 [35], which harbors a gentamicin resistance marker. For conjugal transfers, *E. coli* S-17λ-pir was used as donor strain. After conjugation, clones were selected in M9 mineral medium with biphenyl crystals and gentamicin (10 µg µl^−1^). *P. xenovorans* harboring the plasmid vector was constructed as a control strain.

### Morphological characterization of macrocolonies of *P. aeruginosa* strains

*P. aeruginosa* strains were grown for 7 h; thereafter, the turbidity at 600 nm was standarized. The cultures (10-μL) were plated on tryptone agar (1% w/v) in absence and presence of the antibiotic PMS (600 µM), which generates superoxide. Plates were incubated at room temperature and the growth was monitored using a high-resolution scanner (EPSON, 600 dpi) [24].

### Susceptibility to oxidizing agents

The effect of oxidizing agents on the growth of *P. xenovorans* strains was determined by using a disk diffusion assay [21]. *P. xenovorans* was grown in modified LB medium in absence or presence of antibiotics for 16 h at 30 °C. 100 μl-culture were added to 4 ml of melted soft agar (1% tryptone, 0.5% agar), then plated on 1% tryptone agar plates. For *P. aeruginosa* strains, cells were grown in LB medium in presence of gentamicin for 14 h at 37 °C. Aliquots of oxidizing agents (15 μl) were deposited in 6 mm-diffusion disks (Whatman) at the required concentration onto the agar. *P. xenovorans* plates were incubated for 24 h at 30 °C. The growth inhibition zones were recorded and determined using the ImageJ software (https://imagej.nih.gov/ij/). The diameter of the Petri dish (85 mm) was established as a length reference. The oxidizing agents employed were hydrogen peroxide and paraquat (10 and 20 mM). Paraquat is a redox-cycling compound that constitutes a continuous source of superoxide radical in the cytoplasm, which is able to activate the SoxR transcriptional regulator [23].

### Reactive oxygen species quantification

To measure hydroxyl radical, the fluorescent probe 3′-(*p*-hydroxyphenyl) fluorescein (HPF) (Life Technologies, Carlsbad, California, USA) was employed following manufacturer instructions. *P. xenovorans* strains grown on glucose until early exponential phase were incubated under agitation with the HPF probe in a ratio of 1:1000. After 1 h of incubation, glucose-grown cells were exposed to paraquat or H_2_O_2_ (1 mM). The formation of ROS was monitored for 3 h with agitation at 30 °C. Fluorescence was measured at excitation/emission maxima of 490/515 nm with a fluorescence reader (Tecan Trading AG, Männedorf, Switzerland).

### Isolation of total RNA and quantitative RT-PCR

Total RNA was isolated from LB400 cells grown on glucose (5 mM) until stationary growth phase and incubated for 1 h in absence and presence of paraquat or H_2_O_2_ (1 mM) using a RNeasy mini kit (Qiagen, Hilden, Germany) according to the manufacturer recommendations. DNase treatment was carried out using the TURBO DNA-free Kit (Ambion, LifeTechologies, Carlsbad, CA, USA) to degrade residual DNA. Amplification of the 16S rRNA gene was used as control for DNA contamination using the primers 27f (5′-AGAGTTTGATCMTGGCTCAG-3′) and 1492r (5′-TACGGYTACCTTGTTACGACTT-3′). RNA concentration was quantified using a QubitTM fluorometer (Invitrogen, Carlsbad, California, USA). For RT-qPCR, total RNA (300 ng) was transcribed with First Strand cDNA Synthesis Kit (Thermo Fisher Scientific, Waltham, Massachusetts, USA) according to the manufacturer recommendations. Quantitative RT-PCR reactions were performed using the Kapa Sybr Fast qPCR Master Mix Universal kit (Hoffmann-La Roche, Basel, Switzerland). Primers employed in the analysis are listed in Additional file 1: Table S1. The *gyrB* gene was amplified as a reference gene. Quantitative RT-PCR analysis was performed on Mx3000P qPCR system (Stratagene, Agilent Technologies, Santa Clara, California, USA). To quantify gene expression the method of ∆∆C_T_ was employed [44].

### Proteome analysis

*P. xenovorans* LB400 cells were grown until early exponential phase in mineral M9 medium with glucose (5 mM) as sole carbon source. Cells were washed with a NaCl solution (0.9%) and exposed to paraquat and H_2_O_2_ (1 mM) for 1 h. Cultures without oxidizing agents were used as control. Three biological replicates were performed under these conditions. For the extraction of total proteins, the cells were suspended in one volume of buffer (10 mM Tris-HCI pH 7.4, 5 mM MgCl_2_ and 50 pg mL^−1^ pancreatic RNAase) [70], and sonicated on ice in pulses of 30 s. The cells were centrifuged at 15,022×*g* at 4 °C, followed by protein precipitation with cold acetone. Samples were incubated at − 20 °C for 1 h, centrifuged at 15,022×*g* at 4 °C and the supernatant was carefully discarded. The precipitated proteins were stored at − 20 °C. Protein reduction was performed by adding 5 μl of 200 mM dithiothreitol (DTT) to each sample and incubation at room temperature for 30 min. For the carboamidomethylation, 20 μl of 200 mM iodoacetamide were added to each sample and incubated in darkness for 30 min at room temperature. To remove the remnant of iodoacetamide without reacting, 10 μl of 200 mM DTT were added and incubated in darkness for 30 min at room temperature. Protein digestion was performed using lysil-endopeptidase (LEP) and trypsin. For this purpose, the digestion was performed with LEP for 4 h at 30 °C. Digestion with trypsin was performed for 16 h at 37 °C. Finally, the samples were dried by vacuum centrifugation at Speed-Vac concentrator (Thermo Fisher Scientific, Waltham, Massachusetts, USA).

Desalted peptides were loaded onto a reversed phase column packed in house with ReprosilPur C18 Acqua (1.9 µm diameter; Dr. Maisch, Ammerbuch, Germany) with a length of 30 cm and inner diameter of 75 μm using a nano-liquid chromatography system Easy nLC-1000 (Proxeon Biosystems, Odense, Denmark). Peptides were eluted with a gradient of 2–60% of phase B (0.1% v/v formic acid in acetonitrile) and phase A (0.1% formic acid) for 160 min at a flow rate of 250 nL/min. Injections were made by duplicate.

Spectra were acquired in an LTQ Orbitrap XL mass spectrometer (LC–MS/MS) (Thermo Fisher Scientific Waltham, Massachusetts, USA) by data dependent acquisition (DDA), automatically switching between full scan MS (*m*/*z* 300–2000) at resolution of 60,000 (*m*/*z* 100) and MS/MS with dynamic exclusion of 90 s. The five most intense ions with + 2 and + 3 charges were isolated and fragmented by collision induced dissociation (CID). The mass spectrometer and the gradients in the nLC were controlled by Xcalibur 2.0 software (Thermo Fisher Scientific).

Changes in protein levels were established when changes of ≥ twofold were observed on treated cells compared with untreated cells. The signals corresponding to each peptide obtained from the analysis by mass spectrometry were quantified using the software Mascot Distiller (MatrixScience, Boston, MA, USA) [59]. Subsequently, proteins were identified using *P. xenovorans* LB400 protein databases. Heatmap visualization of proteins classified according to their function was performed using the pheatmap V 1.0.8 R package [37].

### Statistical analyses

Statistical analyses were performed using one-way ANOVA and LSD Fisher test to assess differences in mean values from each experiment. Differences are significant at p < 0.05.

## Results

### *P. xenovorans* LB400 possess genes for a broad antioxidant response

**Fig. 1:**
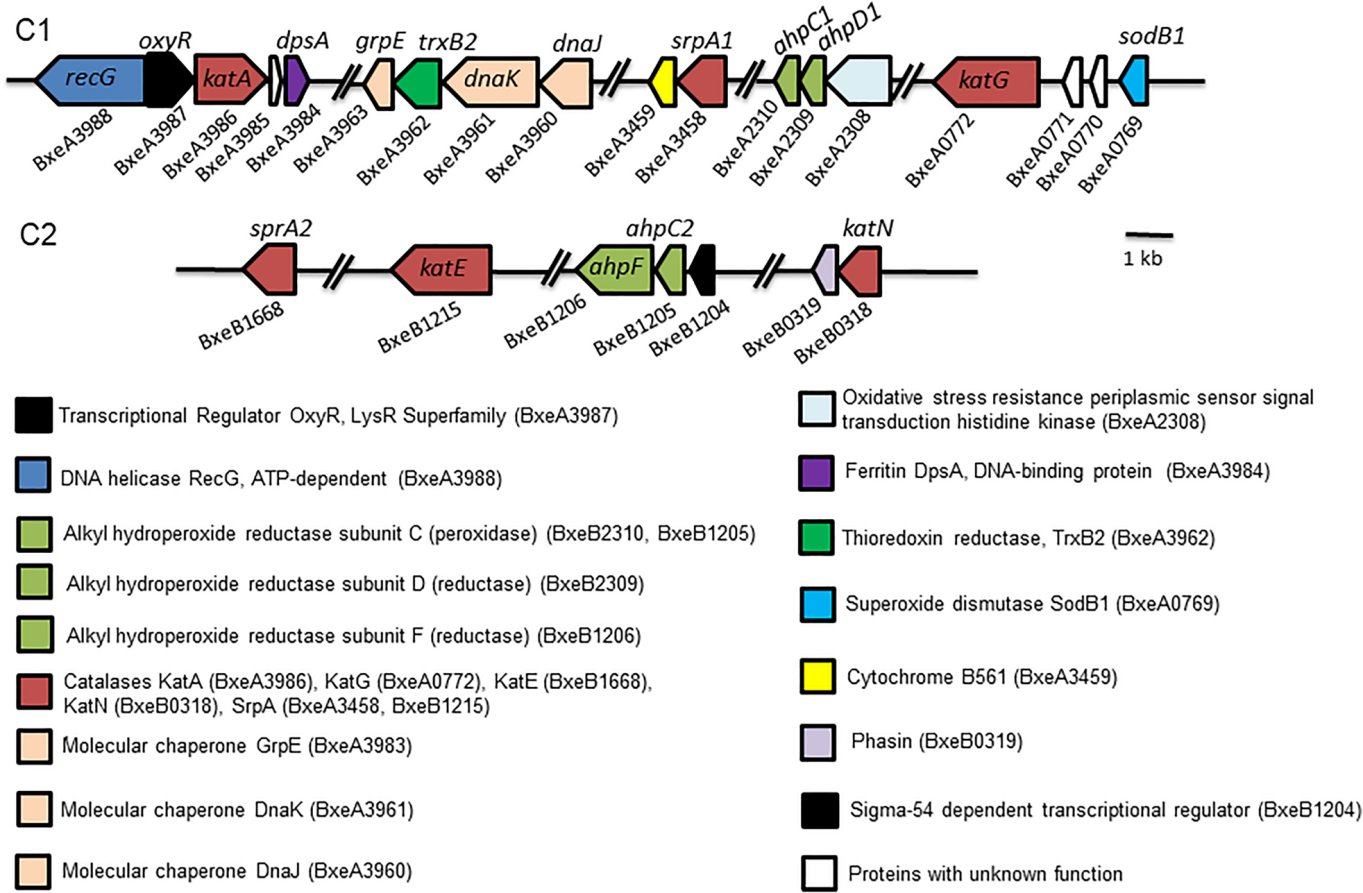
Genomic context of genes involved in oxidative stress response in *P. xenovorans* LB400. C1 and C2 indicate the major and the minor chromosome, respectively

In order to study the oxidative stress response in *P. xenovorans* strain LB400, a search of ortholog oxidative stress genes belonging to the OxyR regulon from *E. coli* that is activated by H_2_O_2_ was performed. Several oxidative stress genes were identified in the genome of strain LB400, including genes encoding 11 alkyl hydroperoxide reductase system subunits (AhpC, AhpD and AhpF), 4 catalases, 3 superoxide dismutases, and a high number of peroxidases and peroxiredoxins (Additional file 1: Table S2). The highest identities (> 80%) were observed with proteins from bacteria of the order *Burkholderiales*. Two subunits of the enzyme alkyl hydroperoxide reductase system, AhpC1 and AhpD1, are encoded by the gene cluster *ahpC1D1* (BxeA2309 and BxeA2310) on the LB400 major chromosome (C1). The minor chromosome (C2) possesses 7 copies of the gene that encodes the enzyme alkyl hydroperoxide reductase subunit D (AhpD), and the gene cluster *ahpC2F* (BxeB1205 and BxeB1206). On the C1 and C2 chromosomes of the strain LB400, the *katA*, *katG*, *katN* and *katE* genes encoding for catalases (one Mn-catalase and three heme-catalases) were identified. The redundancy in the antioxidant systems in strain LB400 is reflected by the high number of copies of oxidative stress response genes. An analysis of the genomic context of the *oxyR* transcriptional regulator gene indicated that it is clustered with other oxidative stress genes, such as the *katA* gene. Upstream of the *oxyR* gene, the *recG* gene (BxeA3988) encoding an ATP-dependent DNA helicase RecG was identified (Fig. 1). Other genes of the LB400 OxyR regulon are the *oxyR* gene encoding the OxyR transcriptional regulator (BxeA3987) and the *gstA1* gene that encodes a glutathione *S*-transferase (BxeA0624).

A search of genes involved in oxidative stress response in *P. xenovorans* LB400, whose orthologous genes are associated to the SoxRS regulon in *E. coli* was performed. The SoxRS regulon in *E. coli* activates the expression of oxidative stress genes, such as *sodA* (superoxide dismutase), *fumC* (oxidative stress resistant fumarase), *acnA* (ROS resistant aconitate hydratase), *fpr* (ferredoxin NADP reductase) and *fldA/fldB* (flavodoxins A and B) [28]. LB400 genome possesses genes encoding the enzyme aconitate hydratase (*acnA1*, *acnA2*), Mn-Fe superoxide dismutase and Cu–Zn superoxide dismutase (*sodB1*, *sodB2*, *sodC*), and a flavodoxin/ferredoxin NADP reductase (*fpr*). The oxidative stress genes identified in the LB400 genome possess high identity with the corresponding proteins of bacteria belonging to the class Betaproteobacteria. LB400 strain possesses the *fumC* gene encoding for fumarate hydratase and the *trxB1* and *trxB2* genes (BxeA3442 and BxeA3962) encoding thioredoxin reductases. Two LB400 genes encode long-chain and short-chain flavodoxins FldX1 and FldX2, which protect cells during oxidative stress [63]. In addition, two homologs of the organic hydroperoxide resistance protein Ohr (BxeB2195 and BxeB2843), regulated in *E. coli* by the organic hydroperoxide resistance transcriptional regulator OhrR, was observed in the LB400 genome. The regulator OhrR was also identified in LB400 strain (BxeB2842). The MerR-type transcriptional regulator SoxR was searched in the LB400 genome. In the LB400 genome, 15 genes encoding MerR-type family regulators were identified. The BxeC1217 (*soxR1*) gene possesses the highest identity with the *soxR* genes from *P. aeruginosa* (31%) and *E. coli* (33%). The *soxS* gene is not present in the LB400 genome, which is expected for non-enteric Proteobacteria. This is in accordance with the observation that SoxR directly activates antioxidant genes in non-enteric bacteria [23, 30]. Other interesting genes involved in stress response are described in Additional file 1: Table S2. The high number of genes identified in the LB400 genome encoding antioxidant enzymes, in some cases with several copies, suggests a broad enzyme repertoire for the cellular response to oxidative stress.

### Phylogenetic analysis of transcriptional regulators

**Fig. 2:**
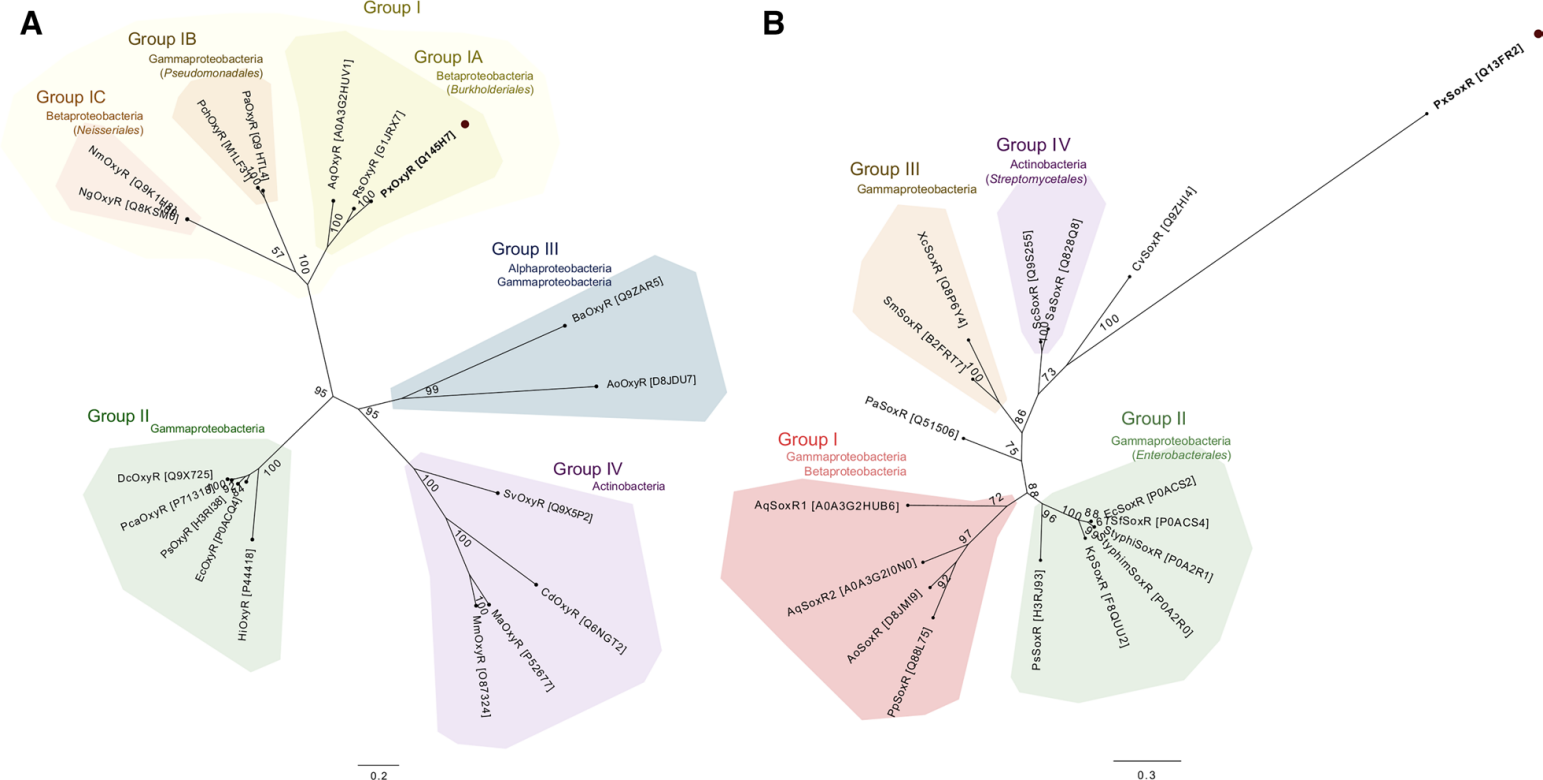
Evolutionary relationships of OxyR and SoxR of *P. xenovorans* LB400 and other bacteria. Mid-rooted Bayesian Inference trees calculated by MrBayes of the OxyR and SoxR regulators with experimental evidence. *P. xenovorans* LB400 OxyR (PxOxyR) and SoxR (PxSoxR) are shown in bold letters and marked with a dark circle. Node values represent the bootstrapping value (%) of the analysis. Each group determined was identified at the class rank level. If the amino acid sequences within each group correspond to the same family rank, the taxon name is included in parentheses. **A** Phylogenetic tree of the H_2_O_2_-sensing transcriptional regulator OxyR homologs. Four major groups were identified, predominantly clustered by taxonomic relatedness. PxOxyR is encompassed within the Group IA, represented by *Burkholderiales*. **B** Phylogenetic tree of the redox-sensitive transcriptional regulator SoxR homologs. Four major groups are identified, including three singleton sequences, not observing a clear taxonomic distribution within the groups. PxSoxR is a singleton closer to the SoxR regulator of *Chromobacterium violaceum* DSM 30191 than to other groups

To investigate the function of the OxyR and SoxR transcriptional regulators in *P. xenovorans* LB400, their phylogenetic relationships were studied. For OxyR analysis, 17 amino acid sequences with experimental evidence in bacteria from Alphaproteobacteria, Betaproteobacteria, Gammaproteobacteria and Actinobacteria classes were used. A Bayesian Inference tree evidenced four main clades within OxyR orthologs, mainly clustered by taxonomic relatedness of the sequences (Fig. 2A). The first clade (group I, Fig. 2A) consists of Betaproteobacteria and Gammaproteobacteria. *P. xenovorans LB400* OxyR (BxeB3987) clustered with the OxyR regulators of other Burkholderiales strains (*Ralstonia solanacearum* K60 and *Alcaligenes aquatilis* QD168) (group IA, Fig. 2A). The second cluster in this clade (group IB, Fig. 2A) is formed by bacteria from Pseudomonadales order, Gammaproteobacteria class (*P. aeruginosa* PA01 and *Pseudomonas chlororaphis* GP72). In the same clade, OxyR of *Neisseria gonorrhoeae* 1291 and *Neisseria meningitidis* MC58 (*Neisseriales* order, Betaproteobacteria) grouped together (group IC, Fig. 2A). The second clade (group II, Fig. 2A) is composed by Gammaproteobacteria *Haemophilus influenzae* KW20 (Pasteurellales order) and Enterobacterales order strains (*E. coli* K12, *Pectobacterium carotovorum* subsp. *carotovorum* AE1202, *Dickeya chrysanthemi* EC16 and *Pantoea stewartii* subsp. *stewartii* DC283). The third clade consists only of OxyR from Actinobacteria, clustered by Mycobacteriales order (*Mycobacterium avium ATCC 19075, Mycobacterium marinum* ATCC 15069 and *Corynebacterium diphtheriae* C7) and *Streptomyces viridosporus* T7A (Streptomycetales order). OxyR of *Brucella abortus* from Alphaproteobacteria class, and OxyR of *Acinetobacter oleivorans* DR1 (Gammaproteobacteria) formed the fourth clade (group IV, Fig. 2A).

For SoxR analysis,16 amino acid sequences with experimental evidence in bacteria from Betaproteobacteria, Gammaproteobacteria and Actinobacteria classes were used. In contrast to OxyR transcriptional regulators that a similar role in diverse bacteria, the SoxR regulators from diverse bacteria have been involved in different functions such as the production and export of redox cycling compounds, the oxidative stress protection, and the development [22, 30, 80]. Therefore, a higher diversity of SoxR regulators is expected due to their association to different functions. The Bayesian inference phylogeny revealed four clades and three singletons, observing an overall robust taxonomic profiling (Fig. 2B). Unlike LB400 OxyR, which clustered with OxyR from other Burkholderiales order, SoxR of strain LB400 (BxeC1217) formed a singleton suggesting a new clade of SoxR regulators. The SoxR of *Pseudomonas putida* KT2440 clustered within the first clade with SoxR of *A. oleivorans* DR1 (Gammaproteobacteria,Pseudomonadales order), and SoxR1 and SoxR2 of *A. aquatilis* QD168 (Betaproteobacteria; Burkholderiales order) (group I, Fig. 2B). In contrast, *P. aeruginosa* PAO1 SoxR (Pseudomonadales order; Betaproteobacteria class) formed a singleton. The second clade is composed of SoxR of the Enterobacterales order (*E. coli* K12, *Salmonella typhi* CT18, *Salmonella typhimurium* LT2, *Shigella flexneri* 301, *P. stewartii subsp. stewartii* DC283 and *Klebsiella pneumoniae* KPBj5 E) (group II, Fig. 2B). SoxR of the Gammaproteobacteria *Stenotrophomonas maltophilia* K279a (Lysobacterales order) and *Xanthomonas campestris* pv. *campestris* P25 (Xanthomonodales order) form the third clade (group III, Fig. 2B). The fourth clade consisted of SoxR of Actinobacteria from the Streptomycetales order, *Streptomyces coelicolor* M145 and *Streptomyces avermitilis* MA-4680 (group IV, Fig. 2B). SoxR of *Chromobacterium violaceum* DSM_30191 (*Neisseriales* order, Betaproteobacteria) formed a singleton.

### P. xenovorans LB400 SoxR has a protective role in P. aeruginosa upon exposure to oxidizing agents

In order to determine the functionality of the *P. xenovorans* LB400 *soxR* gene (BxeC1217), BxeC1217 was complemented in the null mutant strain *P. aeruginosa* ∆*soxR*. Susceptibility assays were performed using the disk diffusion method in the presence of different concentrations of paraquat (10 and 20 mM). Paraquat is a redox-cycling compound that is widely used as herbicide, which is transported inside the bacterial cell and constitutes a continuous source of superoxide radical. Inside the cell, paraquat is reduced by electron donors such as NAD(P)H and is oxidized via the transfer of electrons to the electron acceptor dioxygen, producing superoxide. Redox-cycling agents such as paraquat are able to activate the SoxR transcriptional regulator [23].

**Fig. 3:**
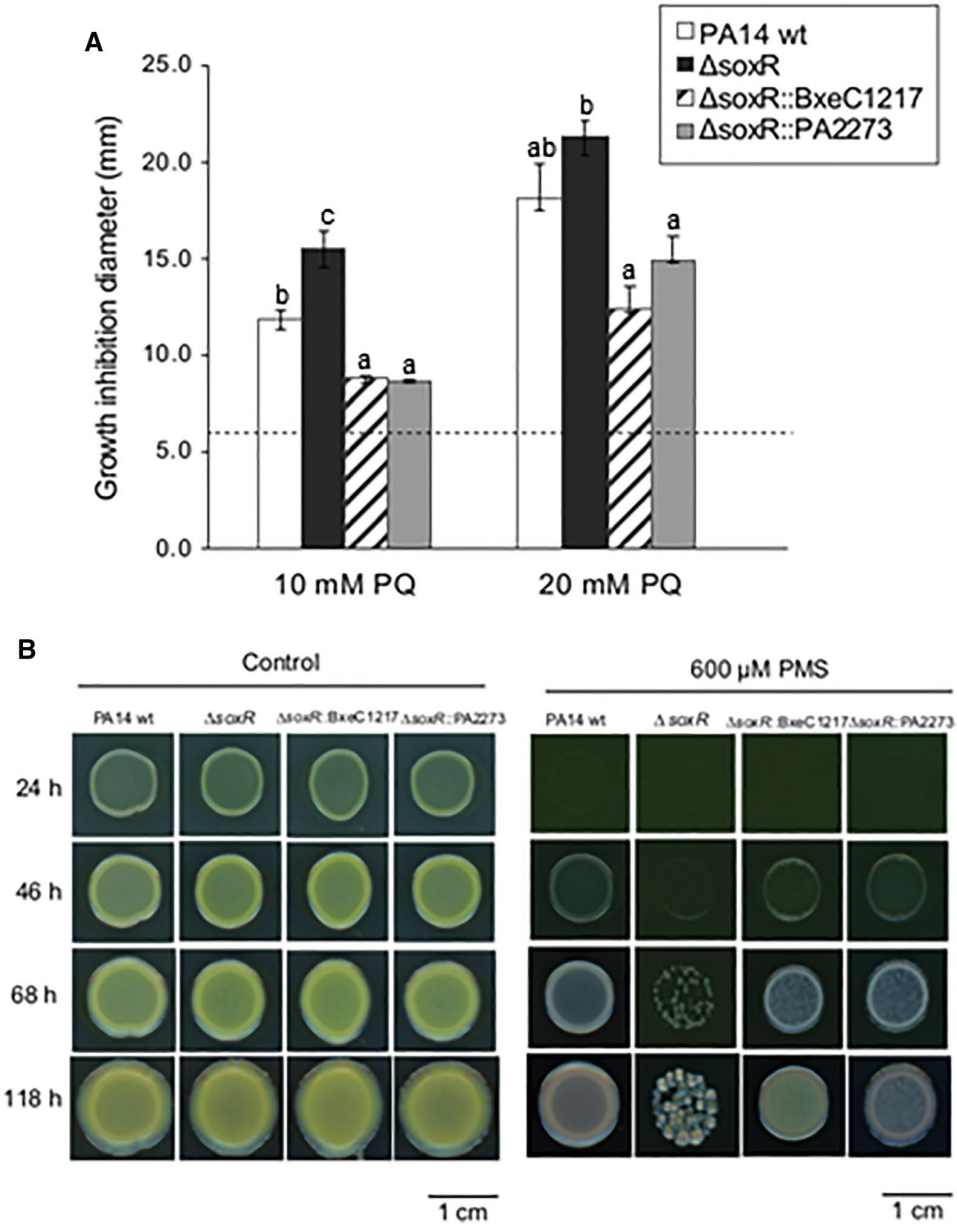
The *soxR* gene of *P. xenovorans* LB400 complements faulty oxidative response in *P. aeruginosa* ∆soxR. **A** Susceptibility assays of *P. aeruginosa* strains PA14 wt, and mutant strains (**∆soxR,** **∆soxR::**BxeC1217 and **∆soxR::**PA2273) exposed to paraquat (PQ). Paper disks soaked with paraquat (10 or 20 mM) were placed on *P. aeruginosa* bacterial lawns. Growth inhibition zones around the disks were recorded after 24 h at 37 °C. The dotted line at 6 mm corresponds to the disk diameter and indicates no detectable inhibition. Inhibition zone values were calculated as the mean ± SD of three independent experiments. Letters under the error bars indicate significant differences between strains in each treatment. **B** Growth of *P. aeruginosa* wt and mutants strains exposed to phenazine methosulfate (PMS) (600 µM). Control cells were grown in absence of PMS. Images are representative of three independent experiments

The complemented strains *P. aeruginosa* ∆*soxR*::BxeC1217 and *P. aeruginosa* ∆*soxR*::PA2273 were less susceptible to paraquat than the mutant strain *P. aeruginosa* ∆*soxR*, with a smaller zone of inhibition (Fig. 3A). These results suggest that LB400 *soxR* gene (BxeC1217) encodes a functional transcriptional regulator SoxR that has a protective effect during oxidative stress induced by paraquat.

To further characterize the role of the *P. xenovorans* LB400 *soxR* gene, the formation of complemented *P. aeruginosa* macrocolonies in presence of the toxic antibiotic PMS was evaluated (Fig. 3B). After 46 h of incubation growth of the null mutant strain ∆*soxR* on plates supplemented with PMS (600 µM) was scarcely observed, while *P. aeruginosa* strains ∆*soxR*::BxeC1217, ∆*soxR*::PA2273 and the wild type strain PA14 grew forming macrocolonies, indicating that BxeC1217 can restore the growth-associated phenotype when exposed to PMS. A yellow-green pigmentation started to appear around 118 h in ∆*soxR*::BxeC1217, which was not observed in PA14 or ∆*soxR*::PA2273 strains (Fig. 3B). The pigmentation suggests that SoxR of LB400 strain regulates also the synthesis in *P. aeruginosa* of the yellow phenazine-1-carboxylic acid and the blue phenazine pyocyanin [21].

### Effect of oxidizing compounds on *P. xenovorans* LB400 growth

**Fig. 4:**
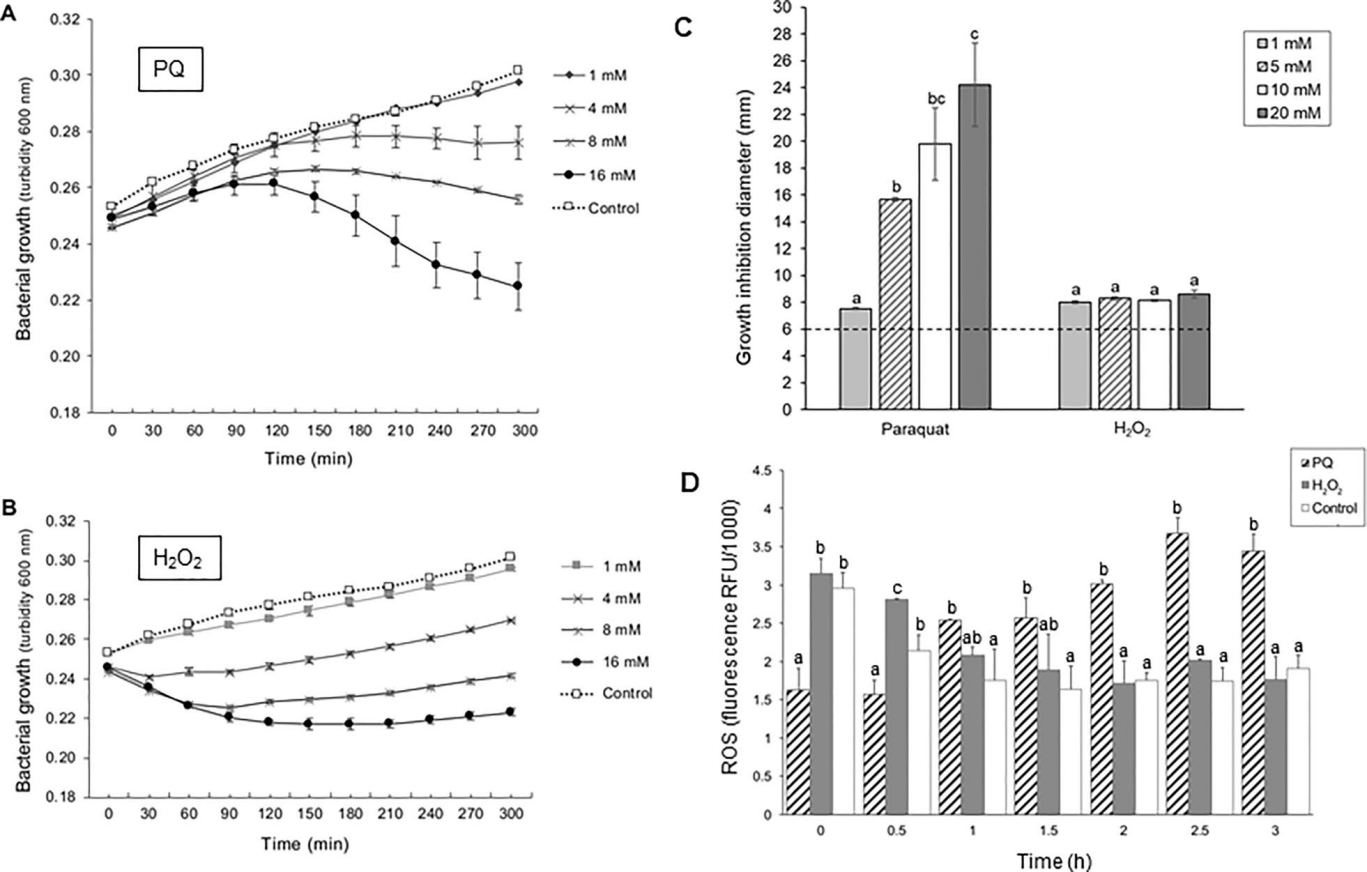
Effects of paraquat and H_2_O_2_ on *P. xenovorans* LB400 growth and ROS formation. Strain LB400 grown until exponential growth phase on glucose was exposed to paraquat (PQ) (**A**) or H_2_O_2_ (**B**). Growth was monitored by measuring turbidity at 600 nm. **C** Paper disks soaked with paraquat or H_2_O_2_ solutions (1–20 mM) were placed on *P. xenovorans* LB400 bacterial lawns. Growth inhibition zones around the disks were recorded after 24 h incubation at 30 °C. The dotted line at 6 mm corresponds to the disk diameter and indicates no inhibition. Letters under the error bars indicate significant differences between each concentration of each oxidizing agent. **D** Exponential glucose-grown cells were exposed to paraquat or H_2_O_2_. After incubation with HPF, fluorescence was monitored at 515 nm (emission) and 490 nm (excitation). Cells incubated in absence of oxidizing agents were used as control. Letters under the error bars indicate significant differences between treatments in each time. All values were calculated as the mean ± SD of three independent experiments

To study the physiological response of the strain LB400 to oxidative stress, growth studies were performed using cells exposed to different concentrations of paraquat and H_2_O_2_. Strain LB400 cells grown on glucose as sole carbon and energy source until early exponential phase were exposed to oxidizing agents in the concentration range from 1 to 16 mM. Growth of strain LB400 was not affected after exposure to paraquat and H_2_O_2_ (1 mM). These results revealed that the oxidizing agents at the concentration 1 mM are not toxic for strain LB400. In contrast, a significant decrease in growth was observed at concentrations ≥ 4 mM of paraquat and H_2_O_2_ (Fig. 4A). Interestingly, an inhibitory effect on growth was observed 30 min upon H_2_O_2_ (4–16 mM) exposure, whereas a negative effect on growth with cell reduction was observed after 2 h exposure to paraquat (4–16 mM) (Fig. 4). Hydrogen peroxide-treated cells (4–16 mM H_2_O_2_) recovered their growth after 120 min. In contrast, paraquat-exposed cells (4–16 mM paraquat) after 120 min reduced progressively the cell turbidity, indicating cell death.

LB400 susceptibility to paraquat and H_2_O_2_ (1, 5, 10 and 20 mM) was assessed during growth in solid medium, using diffusion disks (Fig. 4C). In the case of H_2_O_2_, raising concentrations of the oxidizing agent showed no differences in the inhibition zone (~ 8 mm). An increase in paraquat concentration showed an increase in the inhibition zone within 5 and 20 mM, indicating that strain LB400 is more susceptible to paraquat than to H_2_O_2_.

### *P. xenovorans* LB400 is susceptible to paraquat, which increases intracellular ROS levels

The formation of endogenous ROS in strain LB400 during exposure to paraquat and H_2_O_2_ were studied using a specific probe for the hydroxyl radical. LB400 cells grown to exponential phase on glucose were incubated with a ROS indicator for 1 h. Subsequently, the cells were exposed to paraquat and H_2_O_2_. As shown in Fig. 4D, ROS accumulation was higher in cells exposed to H_2_O_2_ compared to non-exposed cells at short incubation times (0.5 h). An increase in ROS accumulation in paraquat-exposed cells was observed after 1 h incubation, which is later than ROS accumulation in hydrogen peroxide-exposed cells (Fig. 4D).

### *Paraquat and H*_*2*_*O*_*2*_* activate oxidative stress response genes*

**Fig. 5:**
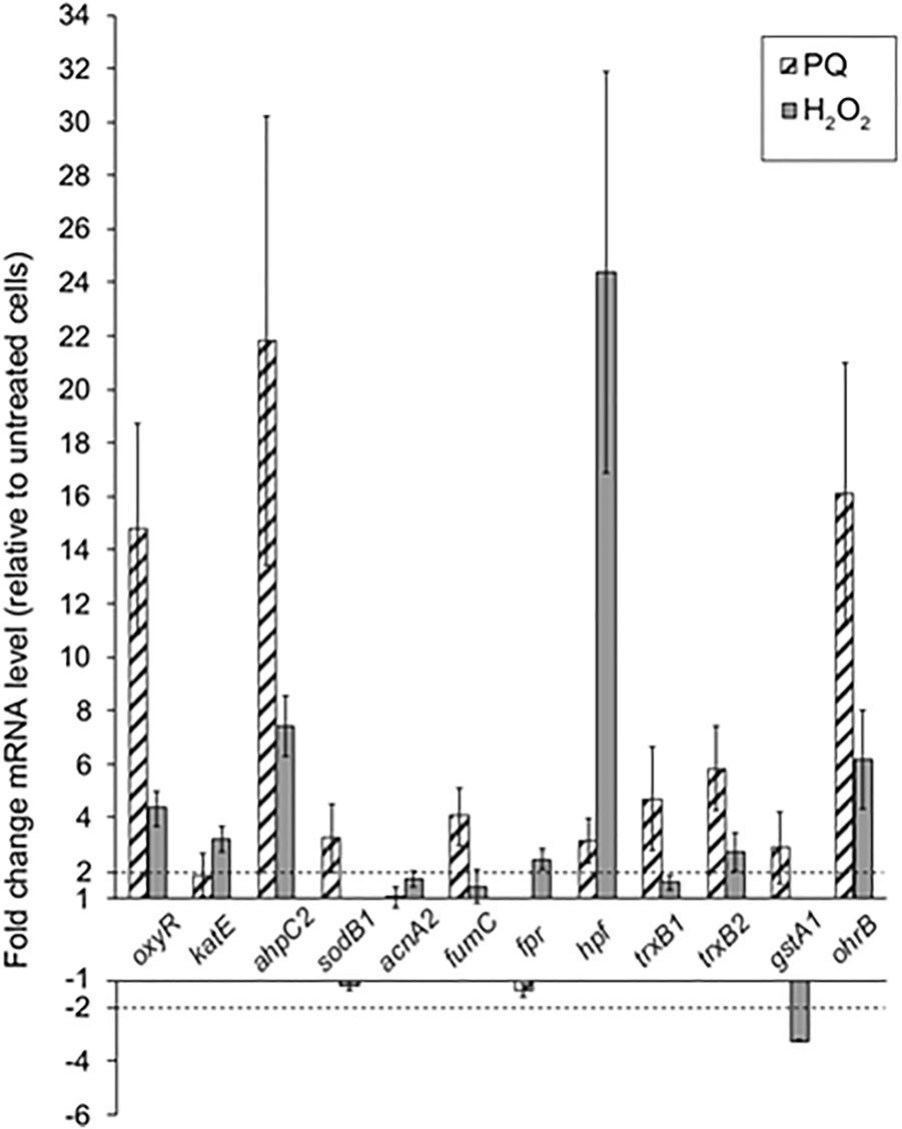
Expression of oxidative stress genes in *P. xenovorans* LB400 upon exposure to paraquat or H_2_O_2_. Expression levels of oxidative stress genes were measured by RT-qPCR after strain LB400 exposure to paraquat (PQ) or H_2_O_2_ (1 mM) for 1 h. The *gyrB* gene was used as the reference gene. Fold-change levels were calculated as the mean ± SD of three independent experiments. The dotted lines indicate the level of expression from which a significant increase (2) or decrease (-2) of expression is observed

Expression of selected genes by RT-qPCR under mild oxidative stress conditions was studied. In order to study the role of selected antioxidant genes, their expression levels were measured in LB400 cells incubated in absence and presence of sublethal concentrations of paraquat or H_2_O_2_ (1 mM). Transcriptional analysis by RT-qPCR of the *oxyR* (BxeA3987), *katE* (BxeB1215), *fumC* (BxeA1038), *acnA2* (BxeB2903), *ahpC1* (BxeA2309), *sodB1* (BxeA0769) genes was performed. Transcriptional expression of additional genes encoding two thioredoxin reductases (*trxB1* and *trxB2*; BxeA3442 and BxeA3962, respectively), a high potential Fe-S protein (*hpf*; BxeA4333), the OhrB protein (*ohrB*; BxeB2843), a flavodoxin/ferredoxin NADP reductase (*fpr*; BxeA4345) and a glutathione *S*-transferase enzyme (*gstA1*; BxeA0624) was also analyzed. In presence of paraquat, the expression of the genes that encode OxyR, AhpC1 and OhrB increased > sixfold compared to non-treated cells (Fig. 5). The *fumC, trxB1* and *trxB2* genes were upregulated in presence of paraquat ≥ fourfold, whereas the *fumC*, *hpf*, and *gstA1* genes increased their expression ≥ twofold. Upon exposure to H_2_O_2_, genes that encode the OxyR transcriptional regulator and the OhrB protein increased > fourfold, while AhpC1-coding gene increased > sixfold. Interestingly, expression of the gene encoding a high potential Fe-S protein (Hpf) increased ≥ 24-fold in presence of H_2_O_2_ compared to the control condition (Fig. 5). In addition, in presence of hydrogen peroxide the transcription levels of the *acnA2*, *katE* genes, and genes encoding a ferredoxin (*fpr*) and thioredoxin reductases (*trxB1* and *trxB2*) increased ≥ twofold. The increase in the expression of antioxidant genes encoding AhpCF and the OxyR transcriptional regulator in the presence of both oxidizing agents, indicate under these oxidizing conditions, a general type oxidative stress response against paraquat and H_2_O_2_.

### *Paraquat and H*_*2*_*O*_*2*_* induce a stress-associated response*

In order to further study the physiological response of *P. xenovorans* LB400 to paraquat and H_2_O_2_, a proteome analysis was performed. LB400 cells grown on glucose until exponential phase were exposed to paraquat and H_2_O_2_ at a sublethal concentration (1 mM) for 1 h. Tables S3 and S4 indicate the significantly induced and repressed (twofold) proteins during exposure to these oxidizing agents.

**Fig. 6:**
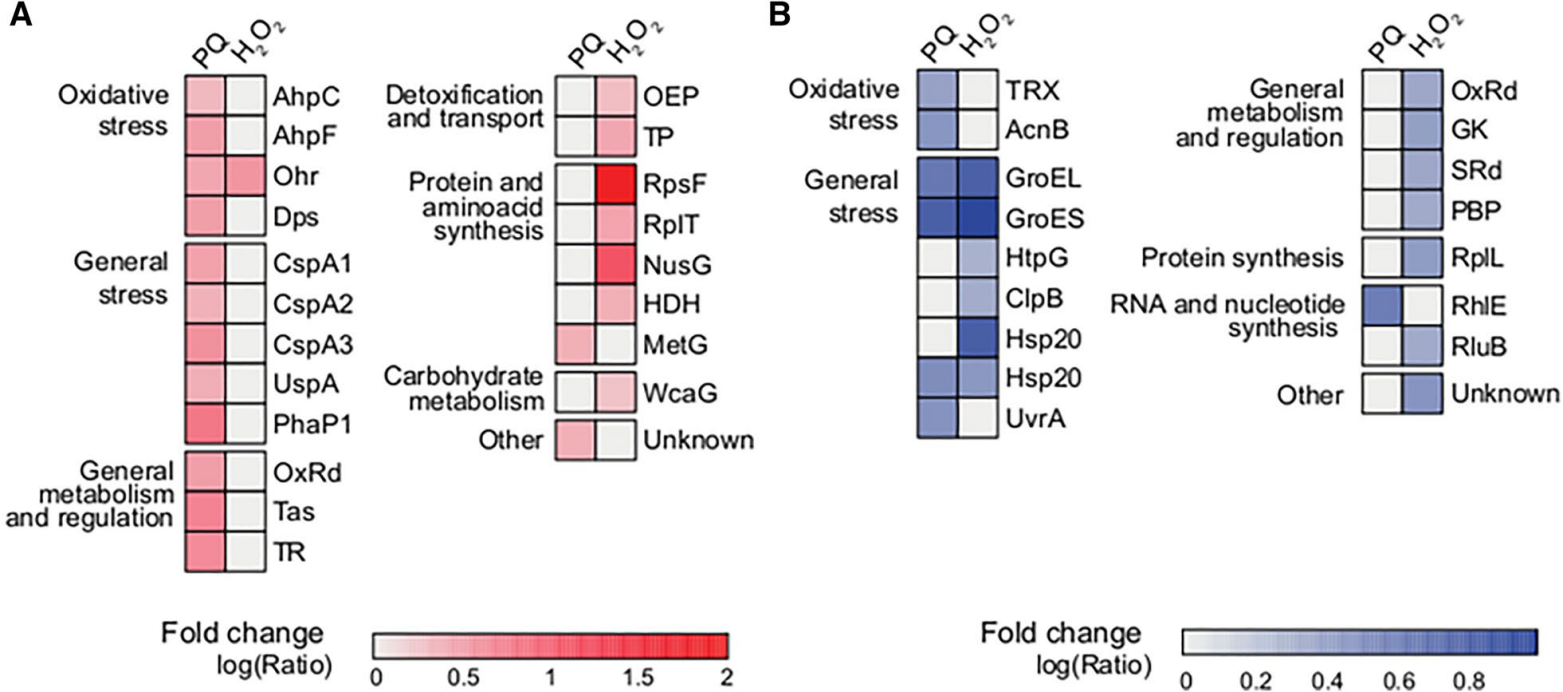
Differentially expressed proteins of *P. xenovorans* LB400 after exposure to paraquat or H_2_O_2_. Heatmap showing upregulated (**A**) or downregulated (**B**) protein levels of *P. xenovorans* LB400 after exposure to paraquat (PQ) or H_2_O_2_ (1 mM) for 1 h compared to untreated cells. Changes in protein levels were established when changes of ≥ twofold were observed on treated cells versus control cells. Red represents higher expression, whereas blue represents lower expression. Three biological replicates were performed. AhpC, alkyl hydroperoxide reductase subunit C2; organic hydroperoxide resistance protein OhrB; Dps, ferritin DPS family DNA-binding protein DpsA. *OxRd* oxidoreductase, *Tas* Tas oxidoreductase, *TR* transcriptional regulator of LysR family, *OEP* outer membrane efflux protein related to copper resistance, *TP* transport protein, *HDH* homoserine dehydrogenase, *GK* glycerate kinase, *SRd* sulphite reductase (NADPH) beta-subunit, *PBP* penicillin binding-protein. Additional file 1: Tables S3 and S4 provide more details of the proteins

During exposure to paraquat (Fig. 6A; Additional file 1: Table S3), the levels of stress-related proteins were increased. Interestingly, the alkyl hydroperoxide reductase AhpC2 and AhpF subunits, and a ferritin protein of the DPS family (DpsA) were induced. The BxeA3984 gene encoding the DpsA protein is present in the *oxyR* gene neighborhood of strain LB400. The organic hydroperoxide resistance protein (OhrB, BxeB2843) was also induced (Fig. 6A; Additional file 1: Table S3). The response of strain LB400 to paraquat included the increase of detoxifying and repair enzymes, along with general stress proteins (Fig. 6A; Additional file 1: Table S3). Induction of the universal stress protein UspA (BxeB0607) was observed during paraquat exposure, as well as three RNA chaperones from the cold-shock DNA-binding protein family (BxeA0430, BxeA0798, BxeB2951). The induction of these chaperones suggests a condition of cellular damage at protein and DNA levels. Interestingly, phasin protein (BxeA1544), a surface protein related to polyhydroxyalkanoates (PHAs) granules, was induced in the presence of paraquat. Paraquat also induced two oxidoreductases (BxeA2478 and BxeA4053), a methylenetetrahydrofolate reductase (MetF) and a transcriptional regulator of the LysR family (BxeA2466), in whose genomic context the genes encoding the CysP, CysT, CysW and CysA subunits of the sulfate/thiosulfate ABC transporter (BxeA2467-BxeA2470) are present. The repression of the stress chaperones GroEL, GroES, HtpG and Hsp20 was observed during exposure to paraquat (Fig. 6B; Additional file 1: Table S4). Moreover, the expression level of the protein UvrA of the UvrABC system (BxeA4108) decreased. An RNA helicase ATP-dependent (BxeA1652) was also downregulated during paraquat exposure (Fig. 6B; Additional file 1: Table S4).

Upon H_2_O_2_ exposure, the organic hydroperoxide resistance protein Ohr (BxeB2843) was strongly induced (Fig. 6A; Additional file 1: Table S3). Two transporters were induced, an outer membrane efflux pump related to copper resistance (BxeB2295) and a transport protein (BxeB1679). Several proteins of the general metabolism were downregulated, including the sulfite reductase beta subunit (sulfur metabolism), a NADH quinone oxidoreductase (oxidative phosphorylation), and a glycerate kinase that participates in serine/glycine/threonine metabolism, glycolipids metabolism and glyoxylate carboxylate metabolism. In addition, the induction and repression of proteins involved in the RNA metabolism, as well as protein synthesis was observed (Fig. 6; Additional file 1: Tables S3 and S4). The induction of the antitermination factor NusG and homoserine dehydrogenase was observed in H_2_O_2_-exposed LB400 cells. The expression levels of the ribosomal proteins S6 and L20 were upregulated, whereas the L7/L12 protein of the 30S and 50S ribosomal subunits were downregulated (Fig. 6; Additional file 1: Tables S3 and S4). The general stress chaperones GroEL, GroES and HtpG as well as the stress chaperones ClpB and HSP20 were repressed.

### OxyR has a protective role in strain LB400 against oxidative stress

In order to study the role of OxyR in strain LB400 during exposure to oxidizing agents, the LB400 *oxyR* gene was overexpressed in *P. xenovorans* (strain pIZ*oxyR*). Susceptibility assays to oxidizing compounds and ROS formation was determined in cells that overexpressed the *oxyR* gene.

**Fig. 7:**
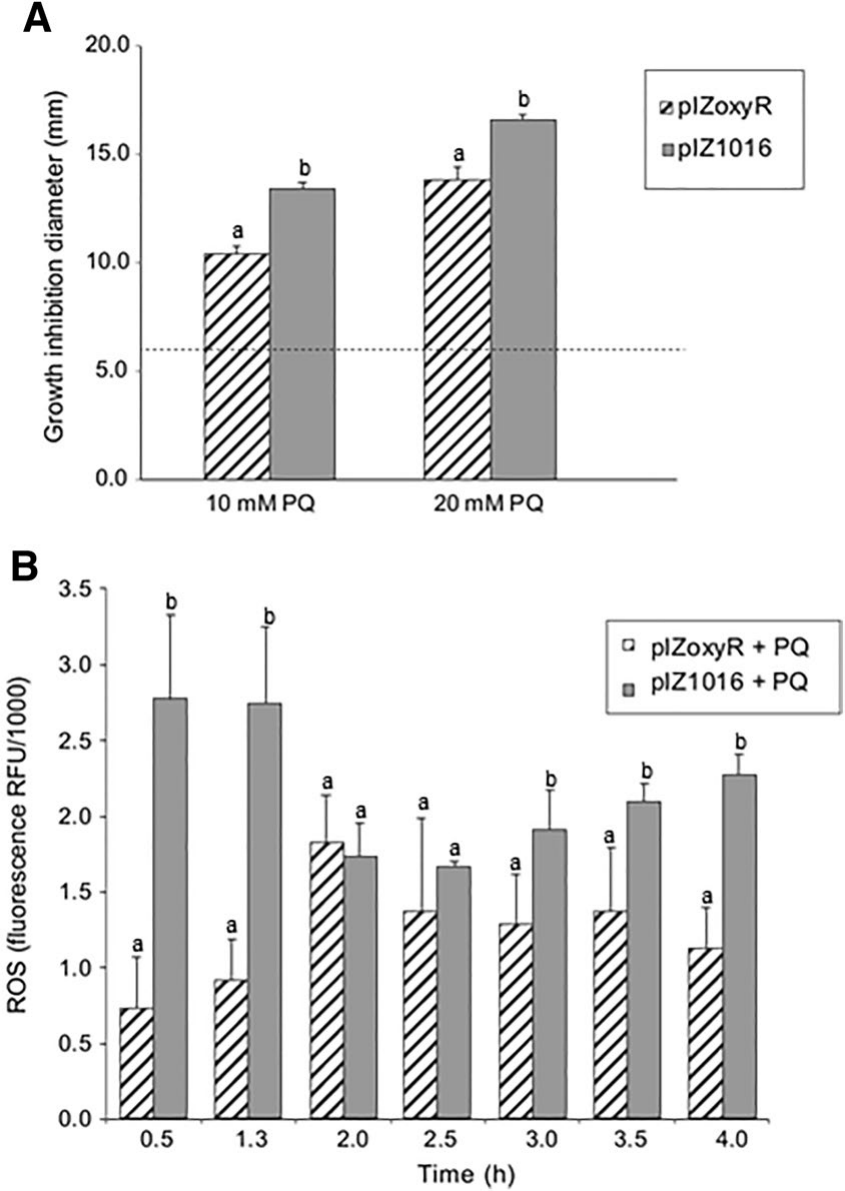
Susceptibility and ROS formation in *P. xenovorans* pIZ*oxyR* exposed to paraquat. **A** Susceptibility assays of *P. xenovorans* strains exposed to paraquat (PQ). Paper disks soaked with paraquat (1–20 mM) were placed on *P. xenovorans* strain bacterial lawns. Zones of growth inhibition around the disks were recorded after 24 h incubation at 30 °C. The diameter of 6 mm corresponds to the disk diameter and indicates no inhibition. Inhibition zone values were calculated as the mean ± SD of three independent experiments. Letters under the error bars indicate significant differences between strains in each treatment. **B** ROS quantification assay. *P. xenovorans* pIZ*oxyR* was grown on glucose (5 mM) until exponential growth phase and exposed to paraquat (20 mM). ROS formation of non-treated cells was also monitored. Fluorescence was monitored after incubation with HPF at 490 nm (excitation) and 515 nm (emission). Fluorescence values were calculated as the mean ± SD of three independent experiments. Letters under the error bars indicate significant differences between strains in each time

Susceptibility assays were performed using different concentrations of paraquat and H_2_O_2_ (10 and 20 mM). As shown in Fig. 7A, strain pIZ*oxyR* was less sensitive to paraquat, showing a slighter zone of inhibition than the control *P. xenovorans* strain (pIZ1016). However, no significant differences upon exposure to H_2_O_2_ were observed between inhibition zones of the pIZ*oxyR* and the reference strain (data not shown).

Subsequently, ROS formation was measured in *oxyR*-overexpressing cells exposed to paraquat and H_2_O_2_ (20 mM). A decrease in ROS accumulation was observed in *P. xenovorans* strain pIZ*oxyR* during exposure to paraquat compared to reference strain (Fig. 7B). However, no significant differences in ROS formation were observed between pIZ*oxyR* and pIZ1016 cells exposed to H_2_O_2_ (data not shown). These results, as well as those observed on susceptibility assays, suggest that OxyR contributes significantly to the oxidative stress response of *P. xenovorans* upon exposure to paraquat.

## Discussion

*Oxidative stress in P. xenovorans LB400.* In this study, we characterized the oxidative stress response of the model aromatic-degrader *P. xenovorans* LB400 to the redox-cycling aromatic compound paraquat and the ROS hydrogen peroxide. The results revealed by genomic, transcriptomic, and proteomic analyses indicate that strain LB400 possess a robust antioxidant enzymatic repertoire. Oxidative stress response in *P. xenovorans* LB400 and other bacteria in presence of aromatic compounds has been previously reported. The aerobic degradation of aromatic compounds is catalyzed by mono-and dioxygenases that uses dioxygen as substrate may produce ROS as side products [33, 61]. During *p*-cymene degradation, LB400 proteome showed an increase of the alkyl hydroperoxide reductase AhpC, the organic hydroperoxide resistance protein Ohr, and the molecular chaperones DnaK, GroEL and ClpB [3]. *p*-Cymene also induced diverse proteins of the energy metabolism, such as aconitase AcnA, succinyl-CoA synthetase, ATP synthase, enolase and pyruvate kinase, which are required for the high-energy demand during the adaptation of glucose-grown LB400 cells to aromatic compounds. (Chloro)biphenyls degradation by *P. xenovorans* LB400 involves oxidative stress, inducing the synthesis of an alkyl hydroperoxide reductase AhpC and molecular chaperones DnaK, GroEL, HtpG and elongation factor G [2, 61]. Chlorobenzoates also generate a general stress response in LB400 cells, inducing the expression of the molecular chaperones DnaK and HtpG [48]. *Burkholderia* sp. BNS showed during phenol catabolism an increase in superoxide dismutase, DNA repair enzymes RecN and RadA, and chaperones Hsp15 and Hsp12 [47]. Fluoranthene catabolism induces expression of catalase and superoxide dismutase in *Mycobacterium* sp. JS14 [42]. *Bacillus subtilis* 168 showed a oxidative stress response to catechol, inducing the alkyl hydroperoxide reductase genes [74]. The addition of antioxidants and the reconfiguration of metabolic fluxes are useful strategies to counteracting oxidative stress of the model aromatic-degraders *P. xenovorans*, *P. putida* and other bacterial strains, which may increase the degradation and bioremediation of persistent organic pollutants [26, 54, 61].

*High redundancy of antioxidant genes.* The broad genetic repertoire of antioxidant genes identified in *P. xenovorans* LB400 (Fig. 1; Additional file 1: Table S2) correlates with its high resistance to hydrogen peroxide and paraquat. LB400 has two clusters encoding two complete alkyl hydroperoxide reductase systems (*ahpC1D1* and *ahpC2F*), and 7 additional *ahpD* gene copies in its genome, which are unusual high *ahp* gene copies in bacteria. In aerobic bacteria, the alkyl hydroperoxide reductase detoxifies organic hydroperoxides and H_2_O_2_ during oxidative stress. In *E. coli, B. subtilis, Salmonella enterica sv. typhimurium* and *Xanthomonas* spp., this enzyme consists of two subunits, a minor subunit with peroxidase activity (AhpC, 22 kDa), and a major subunit with disulfide reductase activity (AhpF, 57 kDa) [62]. AhpC has been reported as the main antioxidant mechanism against endogenous H_2_O_2_ production in *E. coli* [68]. In *S. coelicolor* and *Mycobacterium* species, the *ahpF* gene is absent, whereas downstream of the *ahpC* gene is located the *ahpD* gene, which encodes a thioredoxin-like protein (19 kDa) involved as electron donor in the disulfide reduction of AhpC. Notably, *P. xenovorans* LB400 encodes both AhpCF and AhpCD systems, which has been rarely observed in bacteria. Bioinformatic analyses indicate that *Paraburkholderia hospital* BS001 has genes encoding five AhpC, one AhpD and one AhpF, whereas *Paraburkholderia terrae* possess four AhpC, one AhpD and one AhpF. In *Burkholderia thailandensis*, the AphCD system has been recently characterized [82]. *E. coli* K12, *P. putida* KT2440 and *B. subtilis* 168 possess only one or two copies of AhpC and one AhpF. *S. coelicolor* ATCC BAA-471, *Bradyrhizobium diazoefficiens* JCM 10833 and *Mycobacterium tuberculosis* ATCC 25618 possess one or two copies of AhpC and one or two copies of AhpD.

Four genes encoding catalases (three heme-catalases and one Mn-catalase) are present in the LB400 genome. LB400 KatE possesses a sequence identity of 27.6, 15.3 and 8.8% with LB400 KatA, KatG and KatN, respectively. Uniprot database indicate that the toluene degrader *P. putida* KT2440 possesses three catalases (KatA, KatE and KatG), *E. coli* strain K12 has two catalases (KatE and KatG), and *B. subtilis* 168 possess four catalases (KatA, KatE, KatG and KatX). Three superoxide dismutase-encoding genes were identified in strain LB400. Superoxide dismutase detoxifies superoxide radical, transforming it in H_2_O_2_ and O_2_ [29]. Strain LB400 showed high gene redundancy for the oxidative stress response, redundancy that has been previously described in this bacterium for genes involved in aromatic degradation [14]. A lower redundancy of antioxidant genes (4 *ahp*, 3 kat, 2 *sod*) has been reported in *P. aeruginosa* [55]. The high number of genes encoding antioxidant enzymes in *P. xenovorans* LB400 may contribute to its fitness and tolerance to oxidative stress in environments where bacteria are subjected to a range of adverse environmental conditions.

The transcriptional regulator *oxyR* gene of *P. xenovorans* LB400 grouped together with other antioxidant genes in the *recG-oxyR-katA-dpsA* gene cluster on the major chromosome. A similar gene organization has been observed in Betaproteobacteria and Gammaproteobacteria. This is in accordance with the phylogenetic analysis of OxyR, where LB400 OxyR clustered with OxyR of members from the Burkholderiales order (Betaproteobacteria class). The gene context of the LB400 *oxyR* gene suggests that the *ahpC1D1*, *katA* and *dpsA* genes are activated by OxyR in response to oxidizing compounds, which has been reported in *E. coli* [28]. Interestingly, our study indicates that the LB400 DpsA protein and the *oxyR* gene were induced upon exposure to superoxide-producing compound paraquat. In *B. pseudomallei*, the OxyR transcriptional regulator activates the *katG-dpsA* operon under oxidative stress [32]. The *oxyR-recG* locus is essential in *P. aeruginosa* for the defense to oxidative stress generated by H_2_O_2_ and paraquat [55].

*Functionality of OxyR and SoxR regulators.* In this study, the functionality of OxyR and SoxR regulators in *P. xenovorans* LB400 was demostrated, which showed a protective role against oxidizing agents. The role of the *soxR* gene of *P. xenovorans* LB400 was determined by a complementation assay. In our study, the mutant strain *P. aeruginosa ΔsoxR* complemented with the LB400 *soxR* gene (BxeC1217) was less sensitive to paraquat and phenazine methosulphate, two superoxide-producing compounds. This is particularly interesting considering that SoxR phylogenetic analysis revealed that on one side SoxR of LB400 strain is not closely related to other well characterized SoxR, forming a distinct clade, and on the other side, SoxR showed only 33% identity with its closed bacterial SoxR. The complemented strain *∆soxR::BxeC1217* also changed *P. aeruginosa* pigmentation. These results indicate that the regulator SoxR of *P. xenovorans* LB400 is functional, playing an important role to protect bacteria from superoxide-producing agents (paraquat and PMS), and may activate a specific regulon of not enteric bacteria involved in phenazine production in *P. aeruginosa*. The SoxR regulator is a redox-sensitive transcriptional activator during oxidative stress produced by endogenous or environmental factors. SoxR of *P. aeruginosa* is activated by compounds that produce superoxide, such as paraquat, or also by its own synthesized phenazines [22, 30, 36]. *P. aeruginosa* SoxR activates the PumA monooxygenase and the MexGHI-OpmD efflux pump, which are involved in the modulation of cellular intracellular redox state, and the resistance to exogenous and endogenous phenazines [21, 22, 65, 72]. PumA converts the exogenous phenazine PMS into a highly oxidized compound, a mechanism also proposed for intracellular phenazines produced by *P. aeruginosa* PA14 to control the internal phenazine pool and the redox intracellular state [72]. SoxR from *P. putida, A. oleivorans*, *S. coelicolor*, and *E. coli* are differentially activated by redox-active compounds [34, 42], which could also be the case of LB400 SoxR, inducing an divergent activation of the SoxR regulon in *P. aeruginosa* that may explain ∆*soxR*::BxeC1217 yellow-green pigmentation, probably associated with the synthesis of the yellow phenazine-1-carboxylic acid and the blue phenazine pyocyanin [21].

Notably, we also showed that *oxyR* (BxeB3987) overexpression in *P. xenovorans* conferred higher resistance to the oxidizing agent paraquat (Fig. 7). Paraquat is a continuous source of superoxide radical in the cytoplasm. Paraquat and H_2_O_2_ may activate the OxyR transcriptional regulator in non-enteric bacteria. Interestingly, we observed that paraquat induces stronger the expression of LB400 OxyR than hydrogen peroxide. OxyR activates the H_2_O_2_ scavenging enzymes catalase and alkyl hydroperoxide reductase, which may explain the protective effects observed during paraquat exposure in the *P. xenovorans* strain overexpressing the transcriptional regulator OxyR.

**Fig. 8:**
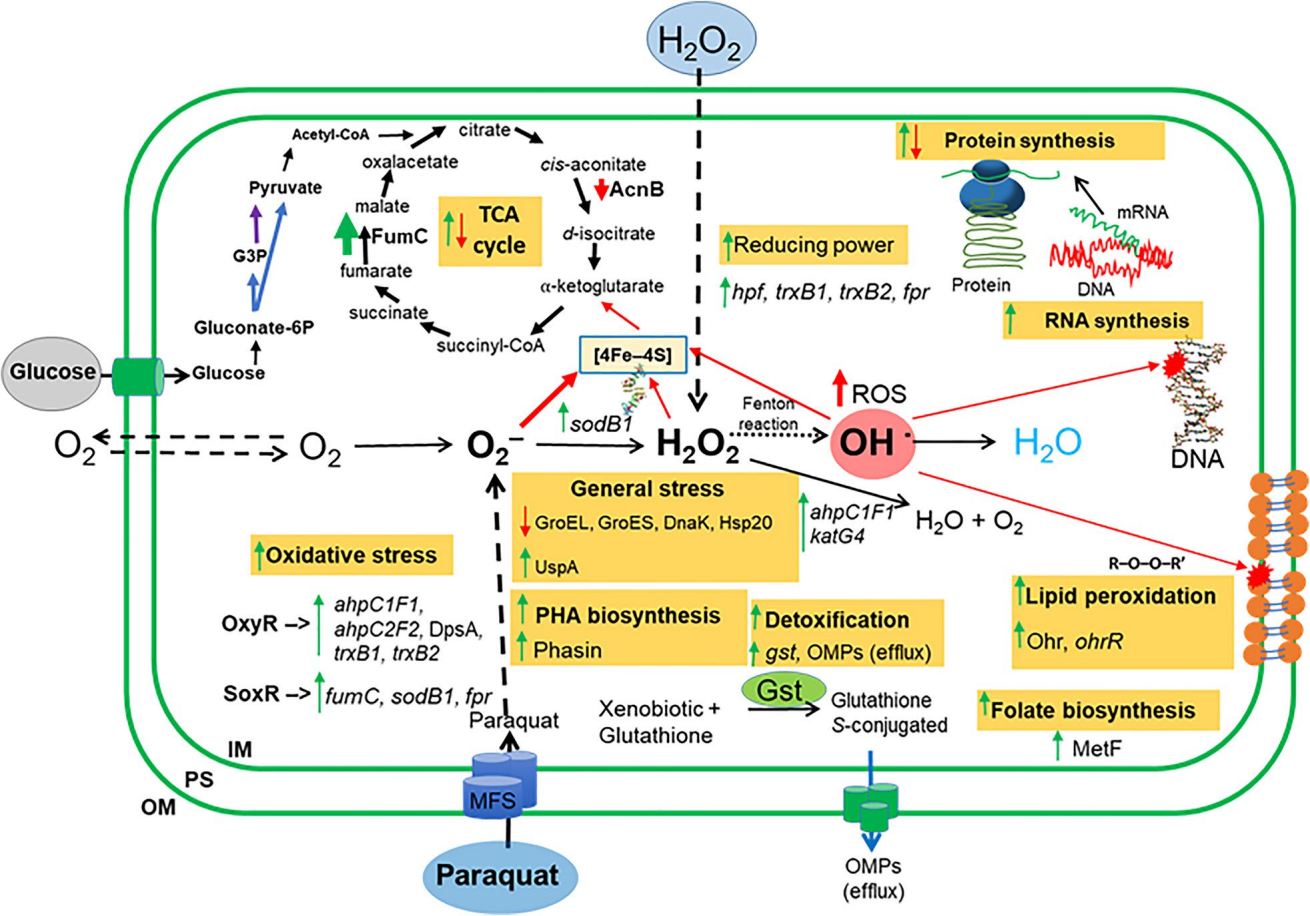
Molecular oxidative stress response of *P. xenovorans* LB400 during exposure to paraquat and H_2_O_2_. Green arrows indicate upregulation of genes and/or proteins, while red arrows indicate downregulation. Dashed arrows indicate the transport of O_2_, H_2_O_2_ and paraquat through the cell membranes. Dotted arrow indicates Fenton reaction, a process during which metal ions (mainly Fe^2+^) react with H_2_O_2_ forming hydroxyl radical. Glucose is converted into gluconate 6-phosphate (gluconate-6P) by glucokinase and the Pentose Phosphate (PP) pathway. Blue arrow indicates the transformation of the gluconate-6P to glyceraldehyde-3-phosphate (G3P) and pyruvate via the Entner–Doudoroff pathway. Purple arrow indicates the conversion of G3P into pyruvate via the lower Embden–Meyerhof–Parnas (EMP) pathway. *OM* outer membrane, *PS* periplasmic space, *IM* inner membrane, *FumC*/*fumC* fumarate hydratase C, *AcnB* aconitate hydratase, *hpf* high potential Fe-S protein, *trxB1* thioredoxin 1,* trxB2* thioredoxin 2,* fpr* flavodoxin/ferredoxin NADP oxidoreductase, *sodB1* superoxide dismutase, *DnaK* GroEL and GroES, molecular chaperones, *Hsp20* heat shock protein, *UspA* universal stress protein, *ahpC1D1* and *ahpC2F* alkyl hydroperoxide reductases, *katE* catalase, *DpsA* ferritin protein of the DPS family, *Gst/gstA1* glutathione *S*-transferase, *Ohr* organic hydroperoxide resistance protein, *OhrR* organic hydroperoxide resistance transcriptional regulator, *MetF* 5,10-methylenetetrahydrofolate reductase

*Response to paraquat and H*_*2*_*O*_*2*_*.* The physiological and molecular response of strain LB400 upon exposure to paraquat and H_2_O_2_ is summarized in Fig. 8. H_2_O_2_ showed after shorter incubation growth inhibition and increased cytoplasmic ROS accumulation probably due to its oxidizing nature, whereas paraquat showed negative effects on growth and increased cytoplasmic ROS formation only after a longer exposure likely because it requires first to generate radicals. Paraquat showed a higher reduction of cell density than H_2_O_2_, probably due to its continuous production of superoxide in the cytoplasm and the membrane diffusion limitation of H_2_O_2_. In *E. coli* the membrane permeability coefficient for H_2_O_2_ is 1.6 × 10^−3^ cm/s, indicating that H_2_O_2_ diffusion inside the cell is limited [69]. This could explain that different concentration of H_2_O_2_ showed no significant differences on growth in liquid medium (Fig. 4B) and in solid medium (Fig. 4C). During the period between 120 and 300 min, the growth rates of cells exposed to different H_2_O_2_-concentration and control condition were similar (~ 10^–4^ h^−1^). In presence of paraquat, higher concentration of the oxidizing agent increased the negative effect on bacterial density, probably to the higher production of superoxide radical inside the cell. Proteomic and transcriptional analyses also indicated that paraquat induced a stronger oxidative stress response than hydrogen peroxide. During exposure to paraquat, the alkyl hydroperoxide reductase system AhpC2F was induced. In addition, the upregulation of the *oxyR* and *ahpC1* genes of strain LB400 was observed in the presence of paraquat and H_2_O_2_, whereas an increase in the *katE* gene expression was observed in cells incubated with H_2_O_2_. The increase of the *ahpC1* gene expression during exposure to paraquat suggests that the superoxide radical produced by paraquat is reduced by superoxide dismutase into H_2_O_2_. These results suggest that *P. xenovorans* displays a broad antioxidant response that includes AhpCF, a catalase and the DpsA protein to protect from the oxidative stress generated by both paraquat and H_2_O_2_, which probably is regulated by OxyR. The hydrocarbon-degrading bacterium A. *aquatilis* QD168 also showed an upregulation of its two *ahpC* gene copies upon paraquat exposure [25]. In bacteria, detoxification of H_2_O_2_ is carried out mainly by the action of alkyl hydroperoxide reductases and catalases [29, 58]. In *E. coli*, the AhpCF enzyme is activated by the regulator OxyR [28]. This indicates that the presence of H_2_O_2_ and paraquat may be triggering the expression of LB400 oxidative stress genes that are probably regulated by the transcriptional regulator OxyR. The *oxyR* gene also increased its expression in strain LB400 in presence of paraquat (> 14-fold) and H_2_O_2_ (fourfold). In *E. coli*, the OxyR regulator activates a specific antioxidant response against H_2_O_2_ with transcription of several oxidative stress genes [28]. The OxyR regulator in *E. coli* is sensitive to H_2_O_2_, whereas in *P. aeruginosa* other non-enteric models the OxyR regulator is activated by different oxidizing agents such as paraquat and H_2_O_2_ [23]. The results of our study suggest that the non-enteric bacterial strain LB400 unfolds a broad stress response against superoxide-producing compound paraquat and H_2_O_2_ mediated by the OxyR regulator.

Other antioxidant genes that may be part of the OxyR regulon in LB400 were upregulated upon paraquat and H_2_O_2_ exposure. The *hpf* gene that encodes for a high potential Fe-S protein, increased > 24-fold in presence of these compounds, whereas the *trxB1* and *trxB2* genes increased twofold. The upregulation of thioredoxin reductases and the Fe-S protein of high potential Hpf in presence of H_2_O_2_ and paraquat, are probably contributing to the LB400 redox state of cells providing reducing power in these oxidizing conditions. Fe-S proteins of high potential are electron-donors that contain a cytochrome tetraheme in their active center [78]. Thioredoxin reductases are widely distributed homodimeric flavoproteins. Bacterial thioredoxin reductases are low molecular weight proteins and their function may vary between organisms. In *E. coli*, thioredoxin reductases transfer reducing equivalents to a wide range of enzymes, which is critical for DNA synthesis and defense against oxidative stress [45]. In addition, they can catalyze the reduction of disulfide bonds of Fe-S cluster proteins [27]. The thioredoxin reductase Trx2, along with the glutathione reductase Grx1, can reduce the disulfide bonds of OxyR protein [28]. At the same time, the OxyR regulator activates the transcription of these and other genes of oxidative stress, which triggers its own self-regulation [45].

Strain LB400 induced the *sodB1* and *fumC* gene expression (fourfold) during paraquat exposure. Paraquat is a continuous source of superoxide radical, which explains the upregulation of the *sodB1* gene in strain LB400. In *E. coli*, genes encoding for the scavenging enzyme SodB1 and ROS-resistant isoform of fumarate hydratase FumC are regulated by SoxRS in response to superoxide [29]. *P. putida* KT2440 exposed to this oxidizing agent also showed an expression increase of the *sodA* and *fumC1* genes [57]. In strain LB400, the ROS-sensitive aconitase AcnB was downregulated in presence of paraquat (Fig. 6). Aconitate hydratase catalyzes the reversible isomerization of citrate and isocitrate via *cis*-aconitate in the Krebs cycle. In *E. coli*, the aconitase AcnB is susceptible to oxidative stress, whereas the aconitase AcnA, which is not dependent on a [4Fe–4S] cluster, plays an important role in survival during oxidative stress [18]. Superoxide radical produced by paraquat could damage [4Fe–4S] dehydratases such as AcnB [79]. This ROS could remove an Fe^2+^ ion from the [4Fe–4S] cluster, inactivating this enzyme [29]. The continuous generation of superoxide radical by paraquat leads to a recurring inactivation of the ROS-sensitive aconitase AcnB. To synthetize an enzyme that could be constantly inactivated represent an unnecessary metabolic burden for the cell, which may explain the downregulation of AcnB when strain LB400 was exposed to paraquat. The results of our study suggest a highly regulated network of key enzymatic components of the oxidative stress response in strain LB400.

Induction of the organic hydroperoxide resistance protein Ohr (BxeB2843) was observed upon paraquat and H_2_O_2_ exposure in strain LB400. A gene encoding the OhrR transcriptional regulator of the MarR family was identified upstream of the LB400 *ohr* gene (BxeB2843). Interestingly, the Ohr protein in *P. aeruginosa* is essential for resistance to organic hydroperoxides but not to H_2_O_2_ or paraquat*,* whereas its induction is independent of OxyR [56]. Exposure of *Cupriavidus pinatubonensis* AEO106 to H_2_O_2_ or sublethal concentrations of copper increased ROS accumulation, and upregulated the Ohr protein [73]. These results suggest that in these bacteria, the Ohr protein plays a key role on stress response during ROS accumulation.

ROS damage biomolecules including proteins [43, 63]. Threonine, arginine, proline, histidine, lysine and tryptophan are the main amino acids targets for oxidation by ROS [41]. LB400 cells exposed to paraquat showed an increase of 5,10-methylenetetrahydrofolate reductase (MetF) gene expression. MetF catalyzes the reduction of 5,10-methylenetetrahydrofolate to 5-methyltetrahydrofolate, a reaction involved in the synthesis of methionine from homocysteine [71]. During H_2_O_2_ exposure, strain LB400 upregulated homoserine reductase, which is involved in the synthesis of threonine from aspartate [81].

Other metabolic processes were triggered upon paraquat exposure. Proteomic analyses showed the upregulation of the phasin PhaP1 (BxeA1544), which is associated to the synthesis of PHA granules. PHAs are linear polyesters produced from sugars and fatty acids, which store carbon and energy in cytoplasmic granules under unbalanced conditions and increase the stress response [1, 19, 76]. The induction of the *phaP1* gene expression during the synthesis of PHA granules by *P. xenovorans* LB400 has been reported [77].

*High resistance to paraquat and H*_*2*_*O*_*2*_**.** Strain LB400 showed a high resistance to the oxidizing agents paraquat and H_2_O_2_ compared to other bacteria. Strain LB400 was capable to resist until 16 mM H_2_O_2_ (Fig. 4B). In contrast, H_2_O_2_ 1.25 mM and 2.5 mM inhibit the growth of *E. coli* and *B. subtilis*, respectively [8]. *P. pseudomonas* MPAO1 growth is inhibited by a H_2_O_2_ (0.6 mM) [20]. *P. xenovorans* LB400 showed much higher survival to paraquat compared to other bacteria. Strain LB400 exposed during 1 h to paraquat (16 mM) showed ~ 96% survival. In comparison, *E. coli* K-12 after 45 min-exposure to paraquat (0.1 mM), showed ~ 75% survival [49]. *P. aeruginosa* PAO1 was also less resistant to 0.2 mM paraquat [46]. *P. xenovorans* strain LB400 exposed to paraquat (20 mM) revealed an inhibition zone of ~ 38 mm^2^, while *Synechocystis* sp. PCC 6803 treated with paraquat (7.8 mM) showed an inhibition halo > 900 mm^2^ [53]. Nevertheless, *B. subtilis* CU1065 is more resistant than strain LB400, showing in presence of paraquat (0.5 M) an inhibition zone of 27 mm^2^ [10]. The reducing power (*e.g.*, NADH, NADPH) generated by the metabolism in the cells is crucial to generate a response to oxidative stress. Interestingly, *P. xenovorans* LB400 and most of the *Burkholderia* sensu lato strains metabolize glucose and other sugars through the Entner-Douderoff (ED) pathway and the pentose phosphate (PP) pathway but not through the classical upper Embden-Meyerhof-Parnas (EMP) pathway because they lack the 6-phosphofructokinase *pfk* gene [1]. The ED pathway and the PP pathway are linked to the lower EMP pathway, producing NAD(P)H reducing power. The activation of the ED route increased the levels of NAD(P)H, which allowed to generate an effective response against the oxidative stress generated by the aerobic metabolism in *P. putida* KT2440 [15, 54]. The induction of NAD reducing enzymes, such as glyceraldehyde-3-phosphate dehydrogenase in *E. coli*, caused an accumulation of NADH via ferredoxin NADP reductase, resulting in the activation of the SoxRS response and an increased tolerance to paraquat [38]. Interestingly, completing the EMP pathway of *P. putida* KT2440 by expressing the *E. coli pfkA*, leads to a decrease in the resistance of this strain to the oxidizing compound diamide [15].

Overall, a broad antioxidant response was observed in *P. xenovorans* strain LB400 against paraquat and H_2_O_2_, in which the OxyR and the SoxR transcriptional regulators and other associated genes play crucial roles.

## Conclusions

Diverse studies have shown that the model aromatic-degrading bacterium *P. xenovorans* LB400 suffers oxidative stress during the catabolism of aromatic compounds. In this study, we have shown that *P. xenovorans* strain LB400 possesses a broad repertoire of genes involved in oxidative and general stress response. In response to the oxidizing aromatic agent paraquat and hydrogen peroxide, strain LB400 displays differential broad oxidative stress responses. Notably, the functionality of OxyR and the SoxR transcriptional regulators were determined through their expression in *P. xenovorans* and *P. aeruginosa* strains. The broad range antioxidant response of *P. xenovorans* LB400, which involves the OxyR and the SoxR transcriptional regulators, may explain in part its metabolic versatility to degrade a wide range of aromatic compounds that causes oxidative stress.

## Supplementary Information

**Additional file 1: Table S1**. Oligonucleotides used as primers for oxidative stress genes of *P. xenovorans* LB400. **Table S2**. Identification of oxidative stress resistance and iron metabolism proteins in *P. xenovorans LB400* genome. **Table S3**. Proteins upregulated in *P. xenovorans* LB400 during exposure to oxidizing agents. **Table S4**. Proteins downregulated in *P. xenovorans* LB400 during exposure to oxidizing agents.

## Data Availability

All data generated or analyzed during this study are included in this published article.

## Abbreviations

OxyR: OxyR transcriptional regulator
SoxR: SoxR transcriptional regulator
ROS: Reactive oxygen species
PQ: Paraquat
PMS: Phenazine methosulfate
HPF: 3′-(*p*-Hydroxyphenyl) fluorescein
DTT: Dithiotreitol
LEP: Lysil-endopeptidase
CID: Collision induced dissociation
