# Supplementary material for: The OxyR and SoxR transcriptional regulators are involved in a broad oxidative stress response in Paraburkholderia xenovorans LB400
Source: Biol Res. 2022 Feb 20;55:7. doi: 10.1186/s40659-022-00373-7 (PMC8859910; doi:10.1186/s40659-022-00373-7)
Supplement: Supplementary file 1 — Additional file 1: Table S1. Oligonucleotides used as primers for oxidative stress genes of P. xenovorans LB400. Table S2. Identification of oxidative stress resistance and iron metabolism proteins in P. xenovorans LB400 genome. Table S3. Proteins upregulated in P. xenovorans LB400 during exposure to oxidizing agents. Table S4. Proteins downregulated in P. xenovorans LB400 during exposure to oxidizing agents. [file 40659_2022_373_MOESM1_ESM.docx]

**Table S1. Oligonucleotides used as primers for oxidative stress genes of *P. xenovorans* LB400**

| **Gene** | **Locus tag** | **Primers** | **Sequence (5'–3')** |
| --- | --- | --- | --- |
| *oxyR* | BxeA3987 | oxyRFwd | GAAGCGTGTTTCGTCAGCCA |
|  |  | oxyRRev | TTCGAGGACACGTTGAGCTT |
| *katE* | BxeB1215 | katEFwd | TCATCGAGGAAGCGGACGAA |
|  |  | katERev | TGTCCGGATTGCGATTGAGC |
| *ahpC1* | BxeA2309 | ahpC1Fwd | GCGTCGACAACGAATTCGTG |
|  |  | ahpC1Rev | TCGATCAGCTCGCCTTTCAC |
| *sodB1* | BxeA0769 | sodB1Fwd | GCGGCTCAAGTGTGGAATCA |
|  |  | sodB1Rev | CTGCGGTCTTGGCGAATTCT |
| *acnA2* | BxeB2903 | acnA3Fwd | GTTACGGTTGCCCGACAGAA |
|  |  | acnA3Rev | GCGTCGACAACGAATTCGTG |
| *fumC* | BxeA1038 | fumFwd | CGTACGAATGGAGCGTGACA |
|  |  | fumRev | ATGAGTTCGGGCGATTGCTT |
| *fpr* | BxeA4345 | fprFwd | GTGACGGTGGGGTAGTACAG |
|  |  | fprRev | CCCGACATTTACGACCGCTA |
| *trxB1* | BxeA3442 | trxB1Fwd | CCCATGAACGCTTCTTCCGA |
|  |  | trxB1Rev | ACCACATTCACACGGCAAAG |
| *trxB2* | BxeA3962 | trxB2Fwd | GACCGGTGTCGATTGGTGTT |
|  |  | trxB2Rev | ATAAACCCGTGCTGCTCGATT |
| *hpf* | BxeA4333 | hpfFwd | CGAGCAAAGTCGACAAAGCG |
|  |  | hpfRev | TATATGCGCTGCACCAACCC |
| *gstA1* | BxeA0624 | gstFwd | GCGACAGCGTATCCACGTATT |
|  |  | gstRev | CTGGTGCCCGAGAATGTCTG |
| *ohrB* | BxeB2843 | ohrFwd | CCCGGCGACAAACTTCATTG |
|  |  | ohrRev | GGCGGTGTCGTCGGATAAT |

**Table S2. Identification of oxidative stress resistance and iron metabolism proteins in *P. xenovorans LB400* genome**

| **Category** | | **Gene** | **Locus tag** | **Protein** | **Aa** | **Organism** | **Access No.** | **Reference** |
| --- | --- | --- | --- | --- | --- | --- | --- | --- |
|  |  |  |  |  |  | **(% identity)** | **(UniProtKB)** |  |
| **Transcriptional regulators** | | | |  |  |  |  |  |
|  | **Oxidative stress** | | |  |  |  |  |  |
|  |  | *oxyR* | BxeA3987 | Hydrogen peroxide-inducible transcriptional activator OxyR | 319 | *Ralstonia solanacearum* K60 (80) | G1JRX7.1 | Cárdenas, 2015 |
|  |  | *soxR* | BxeC1217 | Redox-sensitive transcriptional activator SoxR | 144 | *Streptomyces avermitilis* MA-4680 (31) | Q828Q8.1 | This study |
|  |  | *ohrR* | BxeB2842 | Organic hydroperoxide resistance transcriptional regulator OhrR | 150 | *Xanthomonas campestris pv. Phaseoli (54)* | Q93R11.1 | This study |
|  | **Iron metabolism** | | |  |  |  |  |  |
|  |  | *furA* | BxeA0571 | Ferric uptake regulation protein | 142 | *Cupriavidus metallidurans* CH34 (82) | O30330 | This study |
|  |  |  |  |  |  |  |  |  |
| **ROS scavenger enzymes** | | | |  |  |  |  |  |
|  | **Alkyl hydroperoxide reductases** | | | |  |  |  |  |
|  |  | *ahpC1* | BxeA2309 | Alkyl hydroperoxide reductase, subunit AhpC (peroxidase) | 182 | *Bordetella petrii* DSM12804 (86) | A9II51.1 | This study |
|  |  | *ahpD1* | BxeA2310 | Alkyl hydroperoxide reductase, subunit AhpD (reductase) | 174 | *Rhodospirillum centenum* SW (42) | B6IQZ3.1 | This study |
|  |  | *ahpC2* | BxeB1205 | Alkyl hydroperoxide reductase, subunit AhpC (peroxidase) | 187 | *Salmonella enterica* subsp. *enterica* sv. Typhimurium LT2 (68) | P0A251.2 | This study |
|  |  | *ahpF* | BxeB1206 | Alkyl hydroperoxide reductase, subunit AhpF (reductase) | 530 | *Pseudomonas aeruginosa* PAO1 (73) | Q9I6Z2.1 | This study |
|  |  | *ahpD2* | BxeB0352 | Alkyl hydroperoxide reductase, subunit AhpD (reductase) | 151 | *Pseudoalteomonas atlantica* T6c (52) | Q15SE9.1 | This study |
|  |  | *ahpD3* | BxeB0950 | Alkyl hydroperoxide reductase, subunit AhpD (reductase) | 202 | *Cupriavidus pinatubonensis* JMP134 (84) | Q46Y90.1 | This study |
|  |  | *ahpD4* | BxeB1427 | Alkyl hydroperoxide reductase, subunit AhpD (reductase) | 145 | *C. metallidurans* CH34 (79) | Q1LF94.1 | This study |
|  |  | *ahpD5* | BxeB1537 | Alkyl hydroperoxide reductase, subunit AhpD (reductase) | 187 | *Burkholderia cenocepacia* MC0-3 (76) | B1KB51.1 | This study |
|  |  | *ahpD6* | BxeB1632 | Alkyl hydroperoxide reductase, subunit AhpD (reductase) | 130 | *Azospirillum brasilense* Sp245 (70) | C6K8C8.1 | This study |
|  |  | *ahpD7* | BxeB1911 | Alkyl hydroperoxide reductase, subunit AhpD (reductase) | 130 | *Pseudomonas aeruginosa* 2192 (60) | A0A0B0B821.1 | This study |
|  |  | *ahpD8* | BxeC0103 | Alkyl hydroperoxide reductase, subunit AhpD (reductase) | 211 | *Granulibacter bethesdensis* CGDNIH1 (66) | Q0BPQ1.1 | This study |
|  | **Catalases-peroxidases** | | |  |  |  |  |  |
|  |  | *katG* | BxeA0772 | Heme/peroxidase catalase | 753 | *Paraburkholderia phytofirmans* PsJN (90) | Q13US7.1 | Chain et al., 2006 |
|  |  | *katA* | BxeA3986 | Heme-catalase | 484 | *Bordetella pertussis* Tohama I (73) | P0A323.1 | This study |
|  |  | *katN* | BxeB0318 | Mn-catalase | 294 | *Salmonella enterica* subsp. *enterica* sv. Typhimurium LT2 (75) | Q9KWV1.1 | This study |
|  |  | *katE* | BxeB1215 | Heme-catalase | 707 | *Pseudomonas putida* (67) | P95539.1 | Cárdenas, 2015 |
|  | **Superoxide dismutases** | | |  |  |  |  |  |
|  |  | *sodB1* | BxeA0769 | Superoxide dismutase [Mn-Fe] | 192 | *Ralstonia pickettii* 12D (87) | C6BCI5.1 | This study |
|  |  | *sodB2* | BxeC1205 | Superoxide dismutase [Mn-Fe] | 302 | *Polaromonas* sp. JS666 (64) | Q12AM3.1 | This study |
|  |  | *sodC* | BxeA3479 | Superoxide dismutase [Cu-Zn] | 177 | *C. pinatubonensis* JMP134 (37) | Q46T43.1 | This study |
|  | **Peroxidases-peroxiredoxins** | | | |  |  |  |  |
|  |  | *prx1* | BxeA0492 | Peroxiredoxin sll1621 | 167 | *Synechocystis* sp. PCC 6803 substr. Kazusa (41) | P73728.1 | This study |
|  |  | *prx2* | BxeA3905 | Thioredoxin-dependent peroxiredoxin | 213 | *Aquifex aeolicus* VF5 (45) | O67024.1 | This study |
|  |  | *prx3* | BxeB2802 | Thioredoxin-dependent peroxiredoxin | 212 | *Aquifex aeolicus* VF5 (45) | O67024.1 | This study |
|  |  | *prx4* | BxeB2814 | Thioredoxin-dependent peroxiredoxin | 234 | *Aquifex aeolicus* VF5 (45) | O67024.1 | This study |
|  |  | *bcp* | BxeA2391 | Peroxiredoxin | 153 | *Xanthomonas campestris* pv. *campestris* str. ATCC 33913 (56) | Q8P9V9.1 | This study |
|  |  | *yfeX* | BxeA3833 | Dye-decolorizing peroxidase | 354 | *Escherichia coli* K-12 (36) | P76536.2 | This study |
|  |  | *cpoF1* | BxeB1809 | Non-heme chloroperoxidase | 273 | *Pseudomonas fluorescens* BL914 (69) | O31158.3 | This study |
|  |  | *cpoF2* | BxeB2381 | Non-heme chloroperoxidase | 273 | *Pseudomonas fluorescens* BL914 (68) | O31158.3 | This study |
|  |  | *tpx* | BxeA0528 | Thiol peroxidase | 166 | *Ralstonia solanacearum* GMI1000 (77) | Q8XVP0.1 | This study |
|  |  | *yghU* | BxeA3350 | Organic hydroperoxidase | 235 | *E. coli* K-12 (42) | Q46845.2 | This study |
|  |  | *srpA1* | BxeA3458 | Catalase-related peroxidase | 353 | *Xanthomonas oryzae* pv. oryzae KACC10331 (42) | Q55025.2 | This study |
|  |  | *srpA2* | BxeB1668 | Catalase-related peroxidase | 363 | *Xanthomonas oryzae* pv. Oryzae (48) | Q55025.2 | This study |
|  |  | *osmC* | BxeB1145 | Peroxiredoxin OsmC | 141 | *Escherichia coli* K12 (55) | P0C0L2.1 | This study |
|  |  |  |  |  |  |  |  |  |
| **Thiol-redox homeostasis** | | | |  |  |  |  |  |
|  | **Glutathion S-transferase-Glutaredoxins** | | | |  |  |  |  |
|  |  | *gstA1* | BxeA0624 | Glutathione S-transferase | 206 | *Escherichia coli* K12 (40) | P0ACA1.1 | Cárdenas, 2015 |
|  |  | *gstA2* | BxeA2423 | Glutathione S-transferase | 217 | *Xanthomonas campestris* pv. *campestris* ATCC 33913 (31) | P45875.3 | This study |
|  |  | *gstA3* | BxeA3183 | Glutathione S-transferase | 215 | *Pseudomonas putida* F1 (38) | P82998.2 | This study |
|  |  | *gstA4* | BxeB0925 | Glutathione S-transferase | 203 | *Rhizobium leguminosarum* (67) | Q52828.1 | This study |
|  |  | *gstB1* | BxeA2053 | Glutathione S-transferase | 213 | *E. coli* K-12 (37) | P0ACA7.1 | This study |
|  |  | *gstB2* | BxeA2200 | Glutathione S-transferase | 225 | *E. coli* K-12 (36) | P0ACA7.1 | This study |
|  |  | *gstB3* | BxeB1775 | Glutathione S-transferase | 208 | *E. coli* K-12 (43) | P0ACA7.1 | This study |
|  |  | *grx1* | BxeA0434 | Glutaredoxin (monothiol) | 103 | *Azorhizobium caulinodans* ORS571 (58) | A8I8B7.1 | This study |
|  |  | *grx2* | BxeA4179 | Glutaredoxin (dithiol) | 87 | *Neisseria meningitidis* Z2491 (51) | Q9JVU9 | This study |
|  |  | *gorA* | BxeA1665 | Glutathione peroxidase | 159 | *Ralstonia pickettii* 12D (70) | C6BD65.1 | This study |
|  |  | *arsC1* | BxeB0302 | Arsenate reductase (glutaredoxin) | 140 | *Sinorhizobium meliloti* 1021 | Q92R44.1 | This study |
|  |  | *arsC2* | BxeB3030 | Arsenate reductase (glutaredoxin) | 119 | *Neisseria meningitidis* Z2491 (45) | P63621.1 | This study |
|  |  | *bphK* | BxeC1190 | Glutathione S-transferase (biphenyl pathway) | 202 | *P. xenovorans* LB400 | Q59721.1 | Tocheva et al., 2006 |
|  |  | *yqjG* | BxeA3010 | Glutathionyl-hydroquinone reductase | 347 | *Escherichia coli* K12 (65) | P42620 | This study |
|  | **Thioredoxin reductases-Thioredoxins** | | | |  |  |  |  |
|  |  | *trxA1* | BxeA0743 | Thioredoxin | 282 | *Pasteurella multocida* subsp. *multocida* Pm70 (49) | Q9CM49.1 | This study |
|  |  | *trxA2* | BxeA2362 | Thioredoxin | 108 | *Mycobacterium tuberculosis* ATCC 25618 (92) | P9WG67 | This study |
|  |  | *trxA3* | BxeB1336 | Thioredoxin | 106 | *Chlamydia pneumoniae* J138 | Q9Z7P5.1 | This study |
|  |  | *trxB1* | BxeA3442 | Thioredoxin reductase | 333 | *C. metallidurans* CH34 (82) | Q1LQK6.1 | This study |
|  |  | *trxB2* | BxeA3962 | Thioredoxin reductase | 143 | *R. pickettii* 12D (69) | C6BD28.1 | This study |
|  |  |  |  |  |  |  |  |  |
| **Organic hydroperoxides resistance** | | | | |  |  |  |  |
|  |  | *ohrA* | BxeB2195 | Organic hydroperoxide resistance protein A | 142 | *Xanthomonas axonopodis* pv. *Citri* 306 (66) | P0A0V4.1 | This study |
|  |  | *ohrB* | BxeB2843 | Organic hydroperoxide resistance protein B | 139 | *Xanthomonas axonopodis* pv. *Citri* 306 (61) | P0A0V4.1 | Cárdenas, 2015 |
|  |  |  |  |  |  |  |  |  |
| **ROS resistant isoenzymes** | | | |  |  |  |  |  |
|  |  | *fumC* | BxeA1038 | Fumarate hydratase, class II | 466 | *Ralstonia solanacearum* GMI1000 (77) | Q8XQE8.1 | Cárdenas, 2015 |
|  |  | *acnA1* | BxeB2301 | Aconitate hydratase AcnA | 865 | *Cupriavidus taiwanensis* LMG 19424 (84) | B3R229.1 | This study |
|  |  | *acnA2* | BxeB2903 | Aconitate hydratase AcnA | 905 | *Ralstonia solanacearum* UW551 (83) | A3RVP8.1 | This study |
|  |  |  |  |  |  |  |  |  |
| **Electron transfer proteins** | | | |  |  |  |  |  |
|  |  | *fldX1* | BxeA0278 | Long-chain flavodoxin | 190 | *P*seudomonas *aeruginosa* PA14 (49) | A0A0H2ZDT7 | Rodríguez-Castro et al., 2019 |
|  |  | *fldX2* | BxeB0391 | Short-chain flavodoxin | 175 | *Paraburkholderia phymatum* TM815 (60) | B2JVA6 | Rodríguez-Castro et al., 2019 |
|  |  | *fpr* | BxeA4345 | Ferredoxin NADP reductase | 256 | *Ralstonia eutropha* H16 (74) | YP_728273.1 | This study |
|  |  | *rubA1* | BxeA3342 | Rubredoxin | 63 | *Clostridium pasteurianum* (56) | P00268.2 | This study |
|  |  | *rubA2* | BxeA3813 | Rubredoxin | 56 | *Pseudomonas aeruginosa* PAO1 (62) | Q9HTK8.1 | This study |
|  |  | *rubA3* | BxeB0205 | Rubredoxin | 81 | *Pseudomonas aeruginosa* PAO1 (65) | Q9HTK8.1 | This study |
|  |  |  |  |  |  |  |  |  |
| **Iron metabolism** | | |  |  |  |  |  |  |
|  |  | *dpsA* | BxeA3984 | Ferritin DPS family DNA-binding protein | 165 | *Burkholderia pseudomallei* K96243 (86) | H7C747.1 | This study |
|  |  | *hpf* | BxeA4333 | High potential Fe-S protein | 103 | *Ralstonia solanacearum* GMI1000 (52) | Q8XUU7.1 | This study |
|  |  | *efeN* | BxeB1834 | Deferrochelatase/peroxidase | 435 | *Bacillus subtilis* 168 (44) | P39597.1 | This study |

**Table S3. Proteins upregulated in *P. xenovorans* LB400 during exposure to oxidizing agents.**

|  | | | | | |
| --- | --- | --- | --- | --- | --- |
| **Protein** | | **Locus tag** | **Access No.** | **Ratio**  **(T/C) ^a^** | **Oxidizing**  **agent(s)** |
| **Oxidative stress** | |  |  |  |  |
|  | Alkyl hydroperoxide reductase subunit C2 | BxeB1205 | Q13MD8 | 2.293 | Paraquat |
|  | Alkyl hydroperoxide reductase subunit F | BxeB1206 | Q13MD9 | 3.547 | Paraquat |
|  | Organic hydroperoxide resistance protein OhrB | BxeB2843 | Q13RY7 | 2.990 | Paraquat |
|  |  |  |  | 4.006 | H_2_O_2_ |
|  | Ferritin DPS family DNA-binding protein DpsA | BxeA3984 | Q145H4 | 3.419 | Paraquat |
|  |  |  |  |  |  |
| **General stress** | |  |  |  |  |
|  | Cold-shock DNA-binding protein CspA1 | BxeA0430 | Q13TT6 | 3.383 | Paraquat |
|  | Cold-shock DNA-binding protein CspA2 | BxeA0798 | Q13UV3 | 2.464 | Paraquat |
|  | Cold-shock DNA-binding protein CspA3 | BxeB2951 | Q13S95 | 4.389 | Paraquat |
|  | Universal stress protein UspA | BxeB0607 | Q13KP5 | 2.748 | Paraquat |
|  | Phasin PhaP1 | BxeA1544 | Q13WX8 | 7.107 | Paraquat |
|  |  |  |  |  |  |
| **General metabolism and regulation** | |  |  |  |  |
|  | Oxidoreductase | BxeA4053 | Q145P3 | 3.512 | Paraquat |
|  | Tas oxidoreductase, aldo/keto reductase family | BxeA2478 | Q13ZJ4 | 5.777 | Paraquat |
|  | Transcriptional regulator, LysR family | BxeA2466 | Q13ZI2 | 4.911 | Paraquat |
|  | |  |  |  |  |
| **Detoxification and transport** | |  |  |  |  |
|  | Outer membrane efflux protein related to copper resistance | BxeB2295 | Q13QE7 | 2.157 | H_2_O_2_ |
|  | Transport protein | BxeB1679 | Q13NQ2 | 3.069 | H_2_O_2_ |
|  |  |  |  |  |  |
| **Protein synthesis** | |  |  |  |  |
|  | Ribosomal protein S6 30S, RpsF | BxeA2456 | Q13ZH2 | 133.529 | H_2_O_2_ |
|  | Ribosomal protein L20 50S, RplT | BxeA1374 | Q13WG0 | 3.200 | H_2_O_2_ |
|  | Antitermination factor, NusG | BxeA0301 | Q13TF7 | 15.721 | H_2_O_2_ |
|  | Homoserine dehydrogenase | BxeA2381 | Q13ZA0 | 2.573 | H_2_O_2_ |
|  | 5,10-methylenetetrahydrofolate reductase, MetF | BxeA0180 | Q13T38 | 2.613 | Paraquat |
|  |  |  |  |  |  |
| **Carbohydrate transport and metabolism** | |  |  |  |  |
|  | Nucleoside-diphosphate-sugar epimerase, WcaG | BxeA2389 | Q13ZA9 | 1.963 | H_2_O_2_ |
|  |  |  |  |  |  |
| **Other metabolic processes** | |  |  |  |  |
|  | Non-characterized protein | BxeB1750 | Q13NX3 | 2.613 | Paraquat |
| ^a^ Ratio between obtained values in experimental triplicates of treated cells (T) and control condition (C). | | | | |  |

**Table S4. Proteins downregulated in *P. xenovorans* LB400 during exposure to oxidizing agents.**

| **Protein** | | **Locus tag** | **Access No.** | **Ratio**  **(T/C)^ab^** | **Oxidizing**  **agent(s)** |
| --- | --- | --- | --- | --- | --- |
| **Oxidative stress** | |  |  |  |  |
|  | Thioredoxin TrxA3 | BxeA2362 | Q13Z81 | -2.466 | Paraquat |
|  | Aconitate hydratase B, AcnB | BxeB1533 | Q13NA8 | -2.883 | Paraquat |
|  |  |  |  |  |  |
| **General stress** | |  |  |  |  |
|  | Molecular chaperone GroEL | BxeA4544 | Q13ZW7 | -4.433 | Paraquat |
|  |  |  |  | -6.330 | H_2_O_2_ |
|  | Molecular chaperone GroES | BxeA2610 | Q13ZW8 | -6.507 | Paraquat |
|  |  |  |  | -9.969 | H_2_O_2_ |
|  | Molecular chaperone HtpG | BxeA3329 | Q142T5 | -2.001 | H_2_O_2_ |
|  | Molecular chaperone ClpB | BxeA2374 | Q13Z93 | -2.149 | H_2_O_2_ |
|  | Heat shock protein Hsp20 | BxeB2397 | Q13QP5 | -6.724 | H_2_O_2_ |
|  | Heat shock protein Hsp20 | BxeB2398 | Q13QP6 | -3.255 | Paraquat |
|  |  |  |  | -2.855 | H_2_O_2_ |
|  | UvrABC system, Protein UvrA | BxeA4108 | Q145U8 | -3.042 | Paraquat |
|  |  |  |  |  |  |
| **General metabolism and regulation** | | | | | |
|  | Oxidoreductase quinone NADPH | BxeA3217 | Q142H6 | -2.313 | H_2_O_2_ |
|  | Glycerate kinase | BxeA2298 | Q13Z18 | -2.544 | H_2_O_2_ |
|  | Sulphite reductase (NADPH) beta-subunit | BxeA3664 | Q144B5 | -2.323 | H_2_O_2_ |
|  | Penicillin binding-protein, serine peptidase, MEROPS Family S11 | BxeA4218 | Q146F7 | -2.228 | H_2_O_2_ |
|  |  |  |  |  |  |
| **Protein synthesis** | |  |  |  |  |
|  | Ribosomal protein L7/L12 50S, RplL | BxeA0305 | Q13TG1 | -2.681 | H_2_O_2_ |
|  |  |  |  |  |  |
| **Nucleic acids synthesis** | |  |  |  |  |
|  | RNA helicase (ATP-dependent) | BxeA1652 | Q13X86 | -4.116 | Paraquat |
|  | Pseudouridine synthase | BxeA2823 | Q140U9 | -2.218 | H_2_O_2_ |
|  |  |  |  |  |  |
| **Other metabolic processess** | | |  |  |  |
|  | Signal peptide protein | BxeA2789 | Q140R5 | -2.993 | H_2_O_2_ |

^a^ Ratio between obtained values in experimental triplicates of treated cells (T) and control condition (C).

^b^ Negative values refer to proteins downregulated in treated cells (T) compared to control condition (C).
